# The influence of sample mass (scaling effect) on the synthesis and structure of non-graphitizing carbon (biochar) during the analytical pyrolysis of biomass†

**DOI:** 10.1039/d3ra01911j

**Published:** 2023-05-02

**Authors:** Rahul Ramesh Nair, Patrick A. Kißling, Andreas Schaate, Alexander Marchanka, Madina Shamsuyeva, Peter Behrens, Dirk Weichgrebe

**Affiliations:** a Institute of Sanitary Engineering and Waste Management (ISAH), Leibniz University of Hannover Hannover 30167 Germany nair@isah.uni-hannover.de rahulgenesis@hotmail.com; b Institute of Physical Chemistry and Electrochemistry (PCI), Leibniz University of Hannover Hannover 30167 Germany; c Institute of Inorganic Chemistry (ACI), Leibniz University of Hannover Hannover 30167 Germany; d Laboratory of Nano and Quantum Engineering, Leibniz University of Hannover Hannover 30167 Germany; e Institute of Organic Chemistry and Centre of Biomolecular Drug Research (BMWZ), Leibniz University of Hannover Hannover 30167 Germany; f Institute of Plastics and Circular Economy (IKK), Leibniz University of Hannover Garbsen 30823 Germany; g Cluster of Excellence PhoenixD (Photonics, Optics, and Engineering – Innovation Across Disciplines), Leibniz University of Hannover Hannover 30167 Germany

## Abstract

The porous non-graphitizing carbon (NGC) known as biochar is derived from the pyrolytic conversion of organic precursors and is widely investigated due to its multifunctional applications. At present, biochar is predominantly synthesized in custom lab-scale reactors (LSRs) to determine the properties of carbon, while a thermogravimetric reactor (TG) is utilized for pyrolysis characterization. This results in inconsistencies in the correlation between the structure of biochar carbon and the pyrolysis process. If a TG reactor can also be used as an LSR for biochar synthesis, then the process characteristics and the properties of the synthesized NGC can be simultaneously investigated. It also eliminates the need for expensive LSRs in the laboratory, improves the reproducibility, and correlatability of pyrolysis characteristics with the properties of the resulting biochar carbon. Furthermore, despite numerous TG studies on the kinetics and characterization of biomass pyrolysis, none have questioned how the properties of biochar carbon vary due to the influence of the starting sample mass (scaling) in the reactor. Herein, with a lignin-rich model substrate (walnut shells), TG is utilized as an LSR, for the first time, to investigate the scaling effect starting from the pure kinetic regime (KR). The changes in the pyrolysis characteristics and the structural properties of the resultant NGC with scaling are concurrently traced and comprehensively studied. It is conclusively proven that scaling influences the pyrolysis process and the NGC structure. There is a gradual shift in pyrolysis characteristics and NGC properties from the KR until an inflection mass of ∼200 mg is reached. After this, the carbon properties (aryl-C%, pore characteristics, defects in nanostructure, and biochar yield) are similar. At small scales (≲100 mg), and especially near the KR (≤10 mg) carbonization is higher despite the reduced char formation reaction. The pyrolysis is more endothermic near KR with increased emissions of CO_2_ and H_2_O. For a lignin-rich precursor, at masses above inflection point, TG can be employed for concurrent pyrolysis characterization and biochar synthesis for application-specific NGC investigations.

## Abstract 2

The sample mass during biomass pyrolysis in an thermogravimetric reactor is shown to influence the pyrolysis mechanism and properties of the resultant non-graphitizing carbon. There is an inflection mass scale beyond which this effect is minimal.

## Graphical abstract

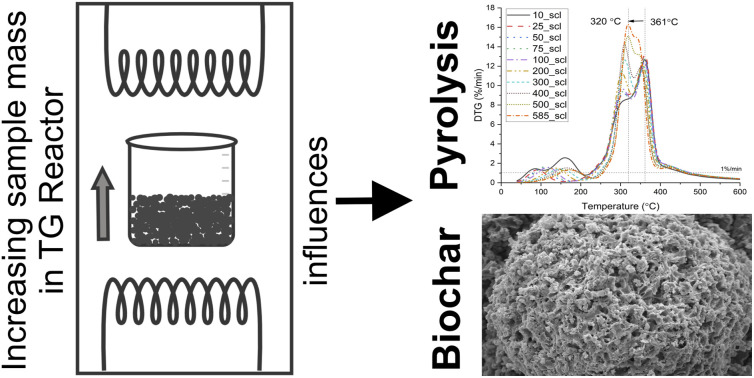

## 1. Introduction

Biochar is the non-graphitizing carbon derived from the slow thermal degradation (pyrolysis) of biomass in an inert or O_2_-deficient environment. The prospective use of this nanoporous material, ranging from carbon sequestration to soil amelioration, has been widely recorded and is an active area of research in environmental and materials science.^1,2^ Due to this broad application scope, slow pyrolysis is widely investigated to optimize the physiochemical properties of the resulting biochar, the emission profiles (greenhouse gases such as CO_2_, CO, NO_*x*_, *etc.*) and the energy or heat of pyrolysis (HoP) required/released during the process. The focus of the existing research has been on pyrolysis characteristics, product (biochar) properties and application-specific experiments such as hybridization, field tests in soil, and adsorption isotherms/kinetics, *etc.*

The common analytical techniques used to elucidate the thermochemical conversion of biomass are (a) thermogravimetric analyzer and/or differential scanning calorimeter coupled to a Fourier transform infrared spectrometer (TG/FTIR and/or TG-DSC/FTIR), (b) pyrolysis – gas chromatography/mass spectrometry (Py-GC/MS), and (c) TG-DSC coupled with a mass spectrometer (TG/MS). Among these, TG-DSC/FTIR is one of the most convenient techniques^3^ due to the simultaneous online measurement of transient mass loss, heat flow, sample temperature, and those evolved volatile molecules with a net change in dipole moment under infrared excitation. Furthermore, a TG-DSC/FTIR is relatively economical and easy to use. Hence, numerous authors^4–7^ have utilized it to investigate the reaction kinetics and thermodynamics of biomass pyrolysis. For the characterization of biochar, custom-built reactors/kilns^8–10^ or muffle furnaces^11–13^ are the most commonly used lab-scale reactors (LSRs) to synthesize biochar in the laboratory. Even for the same organic precursor, this raises the issue of reproducibility of biochar characterization between different research groups and the comparability of the properties of biochar (prepared in LSR) with its pyrolysis characteristics that are studied using the aforementioned analytical methods. For example, most experiments focus either separately on the process properties (such as HoP, reaction kinetics and emissions) using TG-DSC,^14–16^ or on the properties of biochar synthesized by the LSRs.^17–19^ A few experiments^20–22^ have attempted to combine TGA and LSR to simultaneously characterize the process and product during biomass pyrolysis. However, this resulted in discrepancies such as large differences in biochar yield from TG compared to that from LSR as reported by Yu *et al.*^20^ Even when parameters such as feedstock type (particle size and homogeneity), heating rate, and highest treatment temperature (HTT) are kept similar, these inconsistencies arise due to (a) the kinetic regime (sample mass ≲ 15 mg) in analytical TG, which is devoid of the heat and mass transfer effects (HMTE) caused by thermally thick samples that influence the reaction characteristics and secondary tar reactions, (b) the variances between the reactor configurations in LSR and TG.^23^ These reasons cause differences between the properties of biochar generated in TG from that prepared in LSR (as reported by Mašek *et al.*^24^) and their corresponding pyrolysis chemistries.^25^

This has led to the two reasons that motivated this research. First, the question is whether a TG reactor can also be utilized as an LSR for biochar synthesis. If so, then the process characteristics and the properties of synthesized carbon can be simultaneously investigated and correlated. It also eliminates the need for expensive custom LSRs and improves the reproducibility of pyrolysis chemistry in laboratory-scale studies. A steppingstone to answer this question is to understand the effect of starting sample mass (scaling effect) during analytical TG on the pyrolysis characteristics. To the extent of the authors' knowledge, only one other study reports on scaling effects during biomass pyrolysis. This work by Becidan *et al.*^25^ pyrolyzed biomass (coffee waste, fiberboard *etc.*) at two weight scales in a commercially available micro-TG (TA Instruments Q500) and a custom-built macro-TG furnace to show that there are differences in reaction rate and time between the two scales. Thus, this study is limited to generic process differences such as temperature gradient between (only) two scales. The influence trend of this scaling effect on various pyrolysis characteristics (*e.g.*, HoP) in the TG reactor have been left unanswered.

The second reason is the lack of research that explores how the structural properties of biochar NGC synthesized in a TG reactor change when scaling effect is gradually introduced in the pyrolysis that is otherwise performed with chemical kinetics as the rate limiting step. Despite the numerous TG studies on the reaction kinetics of biomass pyrolysis specifically for the field application of biochar,^21,26–28^ none have considered how the properties of the synthesized NGC vary under the influence of mass scaling within the same reactor. Addressing it can also help researchers to develop methods to use TG reactors to simulate different types of slow pyrolysis for tailored biochar design. Hence, the goal of this work is to investigate the trend of influence of scaling effect on the synthesis characteristics and properties of the resultant biochar carbon during pyrolysis in the same reactor.

Herein, this investigation utilizes a TG reactor as an LSR to synthesize biochar from the slow pyrolysis of walnut shell powder (a lignin-rich model substrate) at 10 different mass scales. The pyrolysis is carried out inside a commercially available TG-DSC/FTIR. Then, the structural properties of the resultant biochar carbon are comprehensively investigated through elemental analysis, thermogravimetry, Ar physisorption, ICP-OES, PXRD, Raman spectroscopy and ^13^C-solid-state NMR spectroscopy (ssNMR). The changes in the pyrolysis characteristics (biochar yield, HoP, reaction rate and emission profiles), starting from the mass scale with pure kinetic regime, are also concurrently traced and compared.

## 2. Experimental

Walnut shells (*Juglans regia* L.) were collected, and oven dried at 105 °C for 24 hours and shredded at 8000 rpm in a ZM 200 centrifugal mill (Retsch GmbH, Haan, Germany) using 200 μm blades. The mean of the particle size, bulk density, and thermal conductivity (*λ*) of these powdered walnut shells (WS) were determined to be 129 ± 115 μm, 511.32 kg m^−3^, and 79.82 mW (m^−1^ K^−1^), respectively (details in the ESI†).

### 2.1 Biochar synthesis and pyrolysis characteristics

The TG reactor used was a TGA/DSC 3+ LF/1100 simultaneous thermal analyzer (Mettler Toledo, Ohio, USA) with a protective cell gas flow of 20 ml min^−1^ of N_2_. The microbalance calibration procedure was completed using an aluminum reference sample (99.999% purity, product code – ME 51119701 from Mettler Toledo). The linearity between the reference and sample temperature, and the heat flow were calibrated using reference samples shown in Table 1. The thermal decomposition of WS was investigated using 5 mg of WS pyrolyzed under N_2_ (purity of 99.999% from Linde Gases GmbH) flow of 70 ml min^−1^ from 30 to 1000 °C in 70 μl open crucibles and pierced-lid crucibles (pl_crucibles) made of alumina (material no: 24123, 99.99% purity, Mettler Toledo). Seven linear heating rates (*β*) – 3, 5, 7, 10, 12, 15 and 20 °C min^−1^ – were used to collect thermal analysis data according to the recommendations of the International Confederation for Thermal Analysis and Calorimetry (ICTAC).^29^ Each run was repeated six times and preceded by an N_2_ purge (100 ml min^−1^ for 10 min). The mass and heat flow from each run were blank-corrected using the respective empty crucibles and averaged. Plotted thermograms include weight (%), rate of change of weight or differential thermogravimetry (DTG) curve (% min^−1^), heat flow (W g^−1^) and conversions (*α*) *versus* reference temperature (°C). These data were also used for thermal kinetic analysis.

**Table 1:** Reference samples for the calibration of TGA/DSC3+/LF 1100 °C (Mettler-Toledo GmbH)

Reference sample	Purity	Product code from Mettler Toledo	Certificate number
Indium	>99.999%	ME00119442	9288
Zinc		ME00650014	11056
Aluminum		ME00650016	1501
Gold		ME00650019	24311

For the synthesis of WS biochar at different mass scales (to study the scaling effect), 900 μl alumina crucibles (material no: 51119960, 99.99% purity, Mettler Toledo) and the aforementioned thermal analyzer were used. The WS was weighed and inserted into the crucibles and the crucibles were gently shaken (2–3 times) to ensure uniform distribution of the powder throughout the crucibles (*i.e.*, sample radius ≈ crucible radius). It was linearly heated from 25 to 650 °C at 20 °C min^−1^ and kept isothermal for 5 min under N_2_ purge of 70 ml min^−1^. Ten starting masses (hereafter referred to as scales) of WS were used – 10, 25, 50, 75, 100, 200, 300, 400, 500 and 585 mg. These scales will also be referred to hereafter with the suffix “_scl” such as 10_scl, 25_scl, *etc.* These different scales of WS biochar are collectively referred to as ws_scl. This method for biochar synthesis is summarized in Table 2. The upper limit of sample weight was determined by the maximum volume of WS that a 900 μl crucible can hold. Since the yield of WS pyrolysis is only ∼20%, these trials were repeated at each scale until sufficient biochar was obtained for subsequent analysis. For example, the 10_scl required about 40 trials to complete all analyses.

**Table 2:** Parameters used for the synthesis of biochar from walnut shells (WS) in a TG reactor at different mass scales

Starting sample mass of WS (mg)	Name of the scale of WS (ws_scl)	Linear heating rate (*β*) (°C min^−1^)	Highest treatment temperature (HTT) (°C)	Crucible types	Purge rate of N_2_ during linear heating (ml min^−1^)
10	10_scl	20	650	900 μl alumina open lid	70
25	25_scl				
50	50_scl				
75	75_scl				
100	100_scl				
200	200_scl				
300	300_scl				
400	400_scl				
500	500_scl				
585	585_scl				

For the determination of the heat of reaction (*Q*_r_) during scaling, pyrolysis runs at each scale were repeated 3 to 4 times and data were averaged. Each crucible used in the analysis was blank-corrected to account for any potential effects due to microscopic differences in the physical composition and condition of these crucibles. For comparison, *Q*_r_, was also evaluated with pl_crucibles for all the scales following the same procedure. Evolved gas analysis during scaling was performed using Nicolet iS50 FT-IR (Thermo Fisher Scientific Corporation, Massachusetts, USA) between wavenumber of 4000 and 400 cm^−1^. The temperature of the flow cell and transfer line in the TGA-IR module was maintained at 260 °C during the analysis. The measurement parameters of no. of scans, spectral resolution, signal gain, and optical velocity in the interferometer were 4, 8 cm^−1^, 1, and 0.3165 cm s^−1^, respectively. The FT-IR spectra for each scale were baseline corrected and the chemigram profiles of CO_2_ (2400–2250 cm^−1^), CO (2250–2000 cm^−1^), H_2_O (3990–3400 cm^−1^), CH_4_ (3020–2800 cm^−1^), NH_3_ (980–920 cm^−1^) and mixed organic region (1200–1000 cm^−1^) were generated for semi-quantitative calculation of relative emissions.

### 2.2 Physiochemical characterization of biochar

Mikroanalytisches Laboratorium Kolbe GmbH (Fürth, Germany) performed the elemental analyses of biochar (C, H, N, and O). The measurement uncertainty was ±0.01% for C, H, and N; O was determined from the total by subtraction. In short, biochar samples were weighed and dried overnight at 105 °C. The C, H, and N were measured in a Vario Mikro Cube C, H, N analyzer (Elementar, Frankfurt, Germany). The mean of duplicate measurements was utilized for the evaluation of molar ratios of H/C, O/C and (N + O)/C. To determine the thermal recalcitrance, 5 mg of each sample in 70 μL alumina crucibles was combusted in the TG. The temperature program was a heating rate of 10 °C min^−1^ from 25 to 1050 °C under a synthetic air flow of 60 ml min^−1^. Triplicate measurements were averaged for each sample. The thermal recalcitrance was evaluated as the mass loss ratio during oxidation as shown in eqn (1) where *M* (mg) is the mass of the sample at a given temperature.1
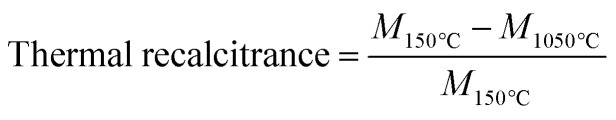


The crystallinity was investigated through powder X-ray diffraction (PXRD) using a Bruker D8 Advance (Bruker, Massachusetts, U.S.A) in reflection mode. It was operated at 20 °C, 40 kV, and 40 mA using Cu-Kα radiation. Each measurement was done in a 2*θ*-range from 5° to 70°, with a step size of 0.010540856°, and 6 s per step, resulting in a total measurement time of 10 h per sample. The biochar samples were transferred into an X-ray amorphous PVC powder carrier and smoothed, to minimize sample displacement. The diffraction patterns were normalized by the max signal intensity for each sample. The database of Powder Diffraction File (PDF 2) 2020 of the International Centre for Diffraction Data (ICDD) was used for pattern identification. Raman spectra were collected with a Senterra microscope (Bruker). The laser had a wavelength of 532 nm with a power of 0.2 mW and a resolution of 3–5 cm^−1^. The integration time was 2 s, and two loops were performed per measuring point. The pore surface area and pore volume were measured through Argon (87 K) physisorption using a 3Flex (Micromeritics, Georgia, USA). Before measurement, the samples were vacuum degassed at 150 °C (ramp rate of 10 °C min^−1^) for 20 h. The physisorption process collected 41 adsorption points and 21 desorption points between 0 and 0.95 *p*/*p*_o_ at 10 s equilibrium time per point.

The ^13^C solid-state NMR (ssNMR) cross-polarization (CP)^30^ and direct polarization (DP) measurements were performed on a 600 MHz SB Bruker Advance III spectrometer (Bruker) equipped with commercial 3.2 mm magic angle spinning (MAS) E-free ^1^H, ^13^C, ^15^N probe head. Typically, about 30 mg of biochar was packed into a thin-walled ssNMR rotor. All experiments were carried out at 16 kHz MAS rate and temperature of 275 K. The pulse lengths of ^1^H and ^13^C 90° were 3.5 μs and 5.0 μs, respectively, and the CP contact duration of 2 and 4 ms were used. A SPINAL64 decoupling^31^ of 70 kHz field strength was applied during acquisition. In total, 32768 scans with 2 s recycle delay were acquired for each CP spectrum. In total, 2048 scans with 15 s recycle delay was acquired for each DP spectrum. The CP spectra were baseline corrected and normalized to the sample mass for further semi-quantitative analysis. The chemical shifts of aryl C, alkyl C and carbonyl C were assigned 110–165 ppm, 50–110 ppm, and 165–200 ppm, respectively.

## 3. Theory and calculation

### 3.1 Characterization of carbon

The powder X-ray diffraction (PXRD) pattern can be used to evaluate *L*_C_ (mean height of graphene crystallite perpendicular to the sheet), and *L*_a_ (mean width of crystallite, parallel to the sheet) according to the Scherrer formula (eqn (2)). This estimates the lattice dimension in the direction perpendicular to the carbon plane where *K* is the shape factor, *λ* is the X-ray wavelength (nm), *β*_*hkl*_ is the full width height maximum (FWHM), *θ* is the Bragg angle and *hkl* are miller indices of the planes being analyzed. The values substituted in eqn (2) for *L*_c_ and *L*_a_ are those at (002) and (100) planes, respectively. The interpretation of the Raman spectra is based on the intensity/height of the dispersive (laser-wavelength dependent) D (*I*_D_) and G band (*I*_G_). The D band signifies the defects and/or edges in graphene-like domains, and G band is from the in-plane bond stretches of sp^2^ carbon atoms (rings and chains). For some carbon materials, additional peaks corresponding to the bands of D* (from sp^3^ bonded carbon atoms), and D** (from sp^2^-bonded carbon) can also be fitted in the Raman spectra.^32^ Thus, the peak fitting procedure can impact the results, especially while estimating the small structural differences in the same type of biochar. Here, after baseline correction between 800 and 2000 cm^−1^, two common peak fitting methods are used (i) 4-peaks fit^33^ with two Lorentz peak at 1350 cm^−1^ (D) and 1590 cm^−1^ (G), and two Gaussian peaks at 1250 cm^−1^ (D*) and 1520 cm^−1^ (D**); (ii) 2-peaks fit^34^ with two Lorentz peaks at D and G bands. Then, *I*_D_/*I*_G_ and FWHM of the G band (FWHM_G_) were evaluated using both fitting methods. The *I*_D_/*I*_G_ ratio is approximately related to the width of the graphene sheet planes (*L*_a_) through the eqn (3). The second-order (2D) Raman spectra (2000–3300 cm^−1^) are related to the stacking along the crystallographic axis, and feature a doublet peak (overtone of the D band, and a D + G band) in highly ordered pyrolytic graphite-like (HOPG).^35^ However, such doublets will not be distinguishable in the 2D region of biochar prepared at low temperatures of 650 °C.2
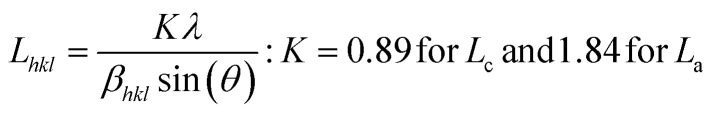
3
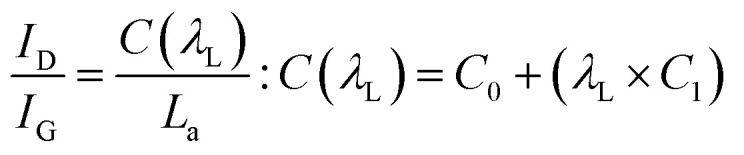


Porous materials are classified as microporous, mesoporous, and macroporous when their internal pore widths are <2 nm, 2–50 nm, and >50 nm, respectively. Pores smaller than 0.7 nm can also be labelled as ultra-microporous. Before activation, biochar usually contains a majority of micropores followed by mesopores.^36^ N_2_ (77 K), Ar (87 K) and CO_2_ (273 K) are some of the adsorbate gases that are commonly used for pore characterization. CO_2_, unless at high pressure, is not sensitive to pores wider than 1 nm. Therefore, CO_2_ as a single adsorbate may not be suitable for the pore characterization of biochar. However, Ar and N_2_ are both suitable for measuring the micro- and mesopore network, with a lower-limit sensitivity of 0.45 nm.^37^ Due to the lack of a quadrupole moment, Argon does not show any specific interactions with the polar functional groups that are usually present in low-temperature (<700 °C) biochar. Thus, Ar is selected as the adsorptive here.^38^ Some samples were measured with N_2_ also to verify the overrepresentation of the surface area due to the quadrupole moment of the N_2_ molecule.^38^ For calculations of pore characteristics – surface area, pore size distribution (PSD) and pore volume – Brunauer–Emmett–Teller (BET) method and non-linear density functional methods (NLDFT) are used. For the BET calculation, only the data points in the range of relative pressure (*p*/*p*_o_) from 0.001 to 0.3 were used, where the upper limit is restricted to the *p*/*p*_o_ above which the *C* parameter (energy of monolayer adsorption) in the BET equation becomes negative.^39^ Pore analysis, namely pore volume and PSD, were performed on the adsorption branch of the isotherms using a 2D-NLDFT model under the assumption of slit-shaped pores and heterogenous surfaces (in terms of energy and geometry).^40^ Finally, the characteristic adsorption energy (*E*_D_) was estimated by the Dubinin–Radushkevich (DR) method.

### 3.2 Pyrolysis characteristics

The thermal decomposition of WS pyrolysis is first studied in the pure kinetic regime where chemical reactions are the rate limiting steps. The mean particle size distribution of the biomass inside the thermogravimetric analyzer should be less than 250 μm (where biot number, *B*_*i*_, <1)^41^ to minimize internal heat transfer limitations (thermal thickness) within the sample particles. Above this limit, the rate of thermal diffusion within a particle is slow enough to become rate limiting. The mean particle size for WS (129 ± 115 μm) lies within this limit and validates the assumption that the sample temperature recorded by the TGA thermocouple is very close to the furnace/reference temperature. The high standard deviation in the particle size is typical for those with non-uniform surface geometry. Except for variables with an equivalent scale of units (*e.g.*, heating rate), all calculations of pyrolysis characteristics uses SI units, unless otherwise mentioned.

Then, the maximum sample mass (*m*_crit_) that can be used in kinetic calculations can be estimated using eqn (4),^42^ where *a* is the thermal diffusivity, *λ* is the thermal conductivity, *c*_p_ is the specific heat capacity, and *ρ* is the bulk density of the biomass, *β* is the heating rate, Δ*T* is the temperature difference through the sample, and *C* is a factor that depends on the aspect ratio (height, *h*_c_ ÷ radius, *r*_c_) of the crucible. When the aspect ratio of 70 and 900 μl crucible is ∼2, then *C* is calculated as in eqn (5). Conventional TG analysis uses masses between 1 and 10 mg. For homogeneous materials, small masses down to 1–3 mg should be sufficient for simultaneous analysis in TG-DSC. However, for an inherently heterogeneous material such as shredded biomass, the results would be representative if higher sample masses could be used per replicate run. However, increasing sample mass may induce temperature gradients within the substrate bulk due to the thermal conduction limitations of the biomass. The *m*_crit_ for WS is 11.34 mg (at *β* = 20 °C min^−1^ and Δ*T* = 1). Hence, 5 mg is selected as the sample mass for kinetic runs, and 10 mg (which is still within the pure kinetic regime) is chosen as the starting mass for scaling experiments.4
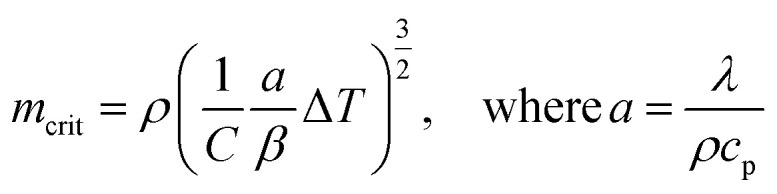
5
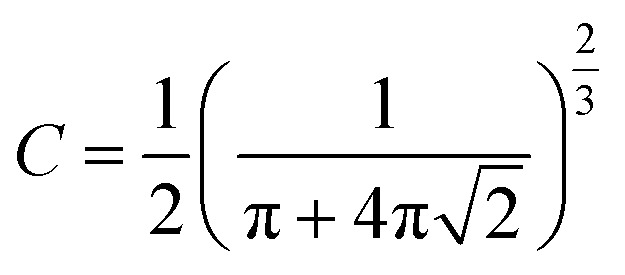


The seven *β* from 3 to 20 °C min^−1^ selected for kinetic runs are according to the recommendations of ICTAC.^29^ Computation of thermal kinetics of WS pyrolysis at different conversion, *α* (eqn (6)), is investigated according to ICTAC,^43^ and has also been extensively discussed by Nair *et al.*^44^ In short, apparent activation energies (*E*_*α*_) at *α* (eqn (6)) are calculated using the non-linear integral isoconversional method to accommodate any non-linearity in sample temperature arising from self-heating during biomass pyrolysis. Then, the pre-exponential factors (*A*_*α*_ or ln *A*_*α*_) are computed using the pseudo kinetic compensation effect (pKCE).^45^ By substituting *E*_*α*_, *A*_*α*_ and the experimentally obtained d*α*/d*T* in eqn (7), the reaction model *f*(*α*) can be found. The eqn (7) is the pressure-independent version of the rate equation at a linear heating rate (*β*) where *R* is the gas constant.6
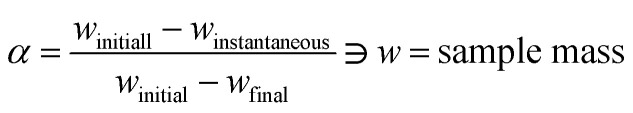
7
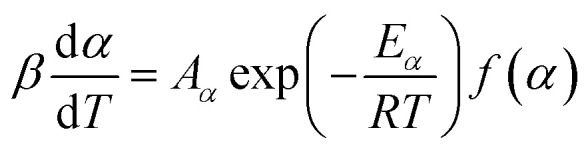
After evaluating the kinetic triplets, the thermodynamic properties at *α*, namely enthalpy change (Δ*H*_*α*_), and Gibbs free energy (Δ*G*_*α*_) are estimated according to eqn (8) and (9), where *A*, *R*, *K*_B_, and *h* are pre-exponential factor, universal gas constant, Boltzmann's constant, and Planck's constant, respectively. *T*_m_ is the peak temperature corresponding to the maximum decomposition rate in the DTG curve.

8 Δ*H*_*α*_ = *E*_*α*_ − *RT*_*α*_

9
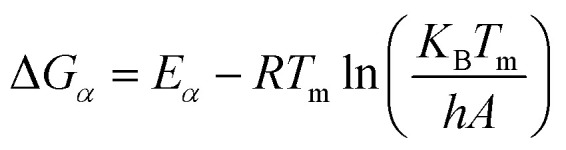


The baseline corrected heat flow measured by the DSC (*Q*_DSC_) in the pure kinetic regime of biomass pyrolysis can be expressed as in eqn (10), where the total of heat of reaction (*Q*_r_), the energy required to heat the biomass (*Q*_b_) and the char (*Q*_c_), radiative heat flow between the char and the furnace (*Q*_rad_), heat loss by the furnace (*Q*_l_). Assuming a thermally insulated furnace and minimal external diffusion limitations (sufficient purging of evolved gases) from the sample surface out of the reactor, *Q*_l_ can be assumed to be negligible. *Q*_rad_ is prominent at higher temperatures and will be negligible in pl_crucibles.^46^*Q*_b_ and *Q*_c_ are evaluated based on *α* according to eqn (11) and (12), respectively. Here, *T*_s_ is the sample temperature, *c*_p,b_ and *c*_p,c_ is the temperature-dependent specific heat capacity of biomass and biochar, respectively. This temperature dependence is shown in Fig. S2, ESI.† As the sample mass increases, the true *T*_s_ at a given point in the sample may differ from the recorded *T*_s_ due to the internal temperature gradient (resulting from the thermal conductivity of the sample) which varies with time and contracting substrate height. It is not possible to correct this thermal lag.^42^ Therefore, both *Q*_DSC_ and *Q*_r_ are reported here.

10 *Q*_DSC_ = *Q*_r_ + *Q*_b_ +*Q*_c_ + *Q*_rad_ + *Q*_l_

11
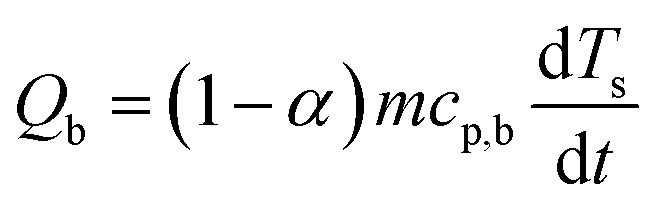
12
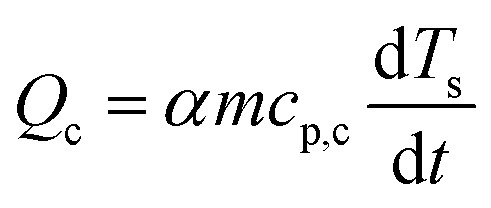


## 4. Results and discussion

### 4.1 Transformation of the biochar carbon with scaling

**Fig. 1:**
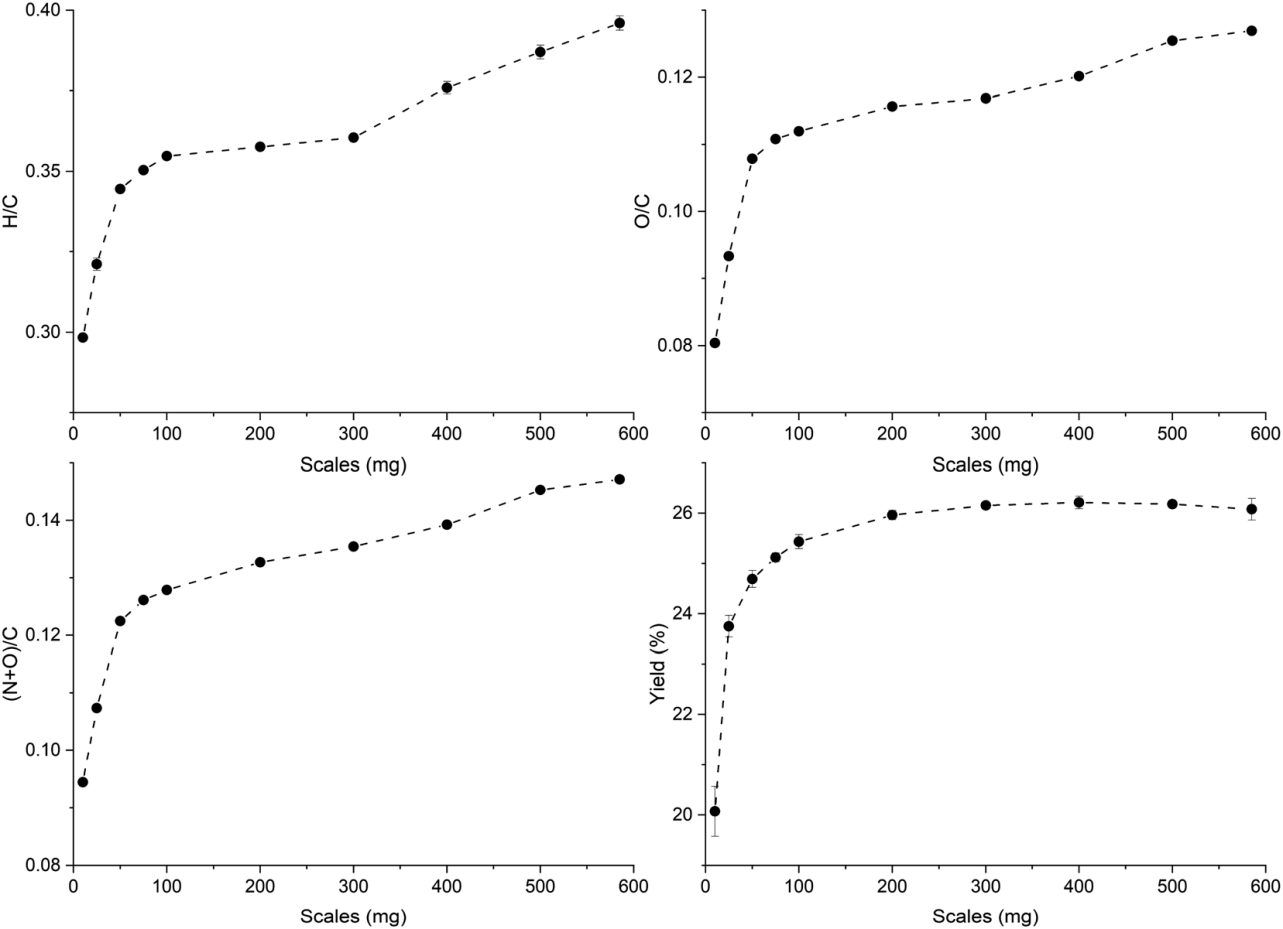
Changes in the molar ratios and the yield (dry-wt%) of the biochar derived from the analytical pyrolysis of walnut shell at different mass scales (mg) in a thermogravimetric reactor.

The trends of H/C and O/C molar ratios of ws_scl are shown in Fig. 1. Usually, these ratios diminish with an increase in pyrolysis HTT as shown by other studies^47,48^ due to the continuous removal of H and O from the biomass with the increase in temperatures. For biochar with less ash content, the low H/C and O/C are considered indicators of the extent of carbonization in them. Since H/C decreases (dehydrative polycondensation) with the formation of larger aromatic clusters, it can be roughly correlated with the aromatic cluster sizes of carbon and its stability in biochar.^49,50^ Using this approximate correlation, increasing sample mass from 10 to 585 mg decreases the biochar stability and shrinks the aromatic cluster size from 6 × 6 to 4 × 4. However, the yield of biochar increases with mass scales and remains fairly constant after 200_scl.

Biochar produced at higher mass scales retains more N and O functionalities, thereby reducing their hydrophobicity. However, the relatively low hydrophilicity of biochar at low mass scales is not a basis for assuming that they “dislike” water molecules, as wettability is also dependent on physical adsorption within the pore network.^51^ The higher retention of N with increasing mass scales may be due to N-heterocyclic compounds (such as pyridine, and pyrrole) from the recondensation of volatile tar compounds on the char surface.^52,53^ The retention of O functional groups makes the biochar produced at higher mass scales relatively acidic. Since the ash content in WS is low (0.8 dry-wt%) to incorporate high oxygen content from inorganic sources,^50^ O/C show a similar trend as H/C. The thermal recalcitrance of ws_scl (Fig. S3, ESI†) also agrees with the trend of H/C; scales below 200_scl have higher oxidative stability from better carbonization. The large std deviations in thermal recalcitrance are due to the possible errors of up to 10% associated with the TGA-based recalcitrance and proximate analyses.^54,55^

In most studies, the pyrolytic conversion of biomass carbon is explained by the increase in graphitization with HTT.^56,57^ This is only an approximate explanation of a rather complex structural transformation of this carbon. At a fundamental level, the graphitization behaviour of carbon has long puzzled researchers.^58^ The seminal works of Franklin,^59^ Kipling *et al.*,^60^ and Harris *et al.*^61^ have proposed various explanations for this process that are still being tested. As an NGC, even at very high temperatures, biochar lacks the ability to form the most thermodynamically stable allotrope of carbon – crystalline graphite. The basic structural unit (BSU) of this disordered carbon lies in a continuum between amorphous carbon (in the parent biomass) and the defected graphite-like material at temperatures approaching 3000 °C. During pyrolysis, the biomass loses moisture and dehydrates up to ∼150 °C. Then, the first carbonization stage (C1) until 350 °C involves the depolymerization of hemicellulose and the crystalline cellulose into randomly disordered carbon. Depending on the type of biomass, amorphous lignin decomposes over a wider range between 200 to 900 °C. Above ∼400 °C, advanced carbonization (C2) of the thermally decomposed lignocellulose is initiated^62^ where the growth, rearrangement and curvature of the BSUs become prominent. This structural arrangement during C2 is influenced by the remaining O and N (heteroatoms) that are covalently bonded to carbon after C1. That is, C2 is influenced by the thermal history during C1. There is considerable evidence^63^ for such an influence of heteroatoms on the C2 stage.

**Fig. 2:**
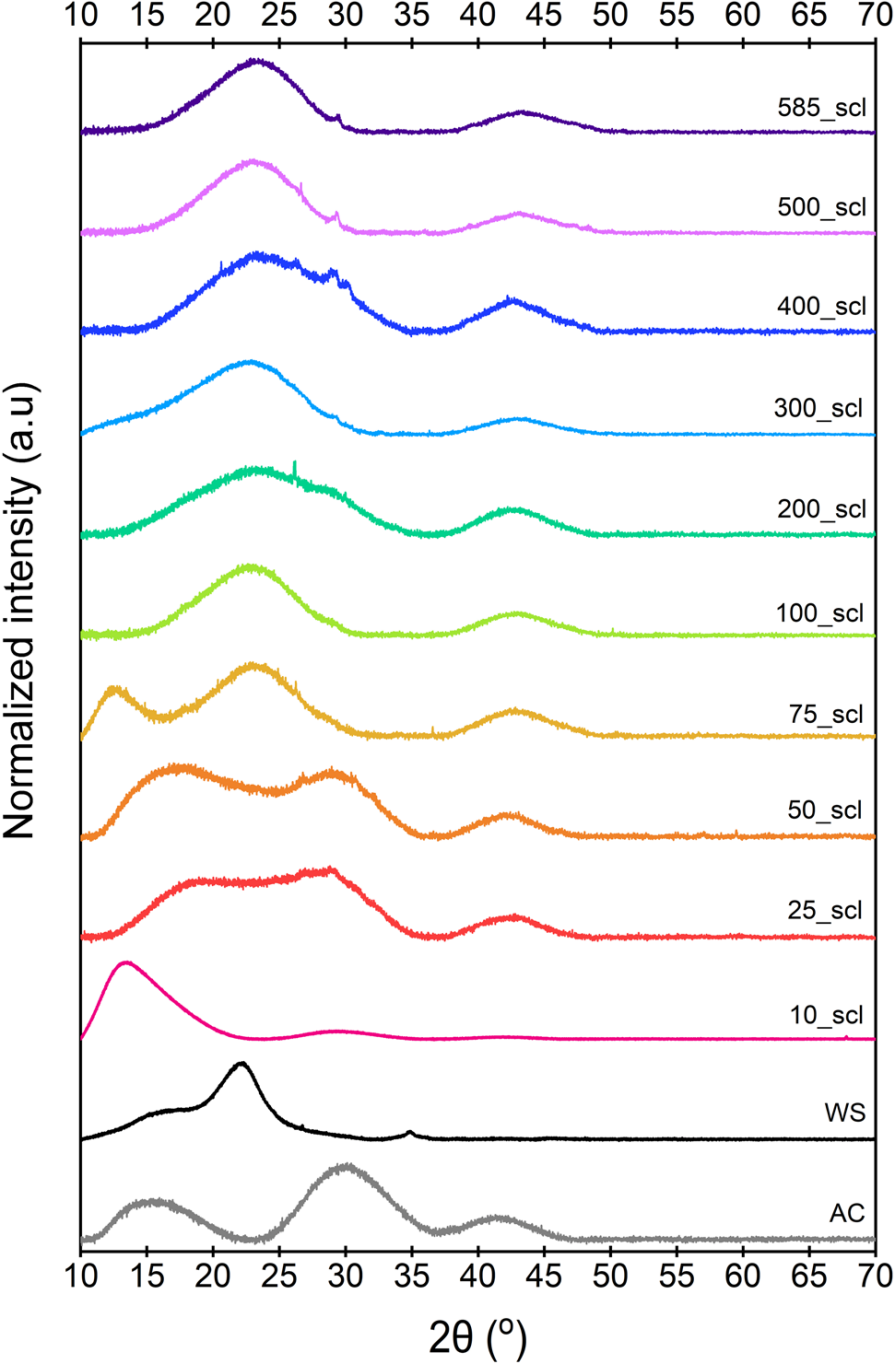
Diffraction patterns of the walnut shell biochar derived at different mass scales at pyrolysis temperature of 650 °C. WS is the parent biomass, while AC is an activated carbon used for reference. 10_scl to 585_scl represents mass scales from 10 to 585 mg, respectively.

The broad diffraction peaks of ws_scl, WS, and a reference activated carbon (AC)^64^ obtained from the PXRD patterns (Fig. 2) show very poor evidence for crystallinity compared to synthetic graphite. The diffraction peak at 16° (2*θ*) may be from clusters of sp^2^ amorphous carbon (s-ac). This is explicitly visible until 75_scl (scales having low H/C). Beyond this, the peak combines with the graphitic basal plane at (002). The (002) reflection (due to stacking of BSU) for the AC reference and scales until 50_scl is at ∼29° (2*θ*). Then, the reflections shift to ∼22°. Some studies^62,65^ have attributed this shift in (002) reflections to lower scattering angles as evidence of decreasing graphite order. This is possible here when compared with the H/C ratios. However, this may not be the only reason as there is no noticeable blue shift of the G band of the Raman spectra (discussed later) at these scales. The (002) shifts may also be due to either the increased thermal strains at scales above 75_scl^66^ or the alignment of the sample with respect to the diffractometer.^67^ Both ws_scl and AC show (100) reflection at ∼42° (2*θ*) characterizing the in-plane growth of BSU (*L*_a_). Even though the biochar yield is higher with increasing scales, this does not translate to lateral growth of BSU in them.^68^ The mean *L*_a_ for ws_scl obtained from eqn (8) is only ∼2.3 nm, which is close to the limit of the applicability of the Tuinstra–Koenig relation (eqn (3)).^69^ Therefore, the *I*_D_/*I*_G_ from Raman analysis must be directly proportional to *L*_a_ for ws_scl.

**Fig. 3:**
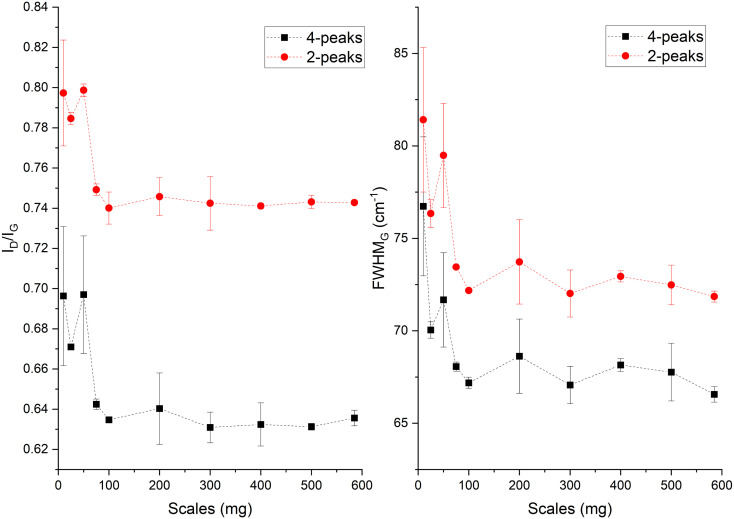
The *I*_D_/*I*_G_ ratio and full width half maximum of the G band (FWHM_G_) evaluated from the Raman spectra of the WS biochar derived at different mass scales during analytical pyrolysis in thermogravimetric reactor. The terms 4-peaks and 2-peaks represents the two fitting methods used in the analysis of the Raman spectra.

**Fig. 4:**
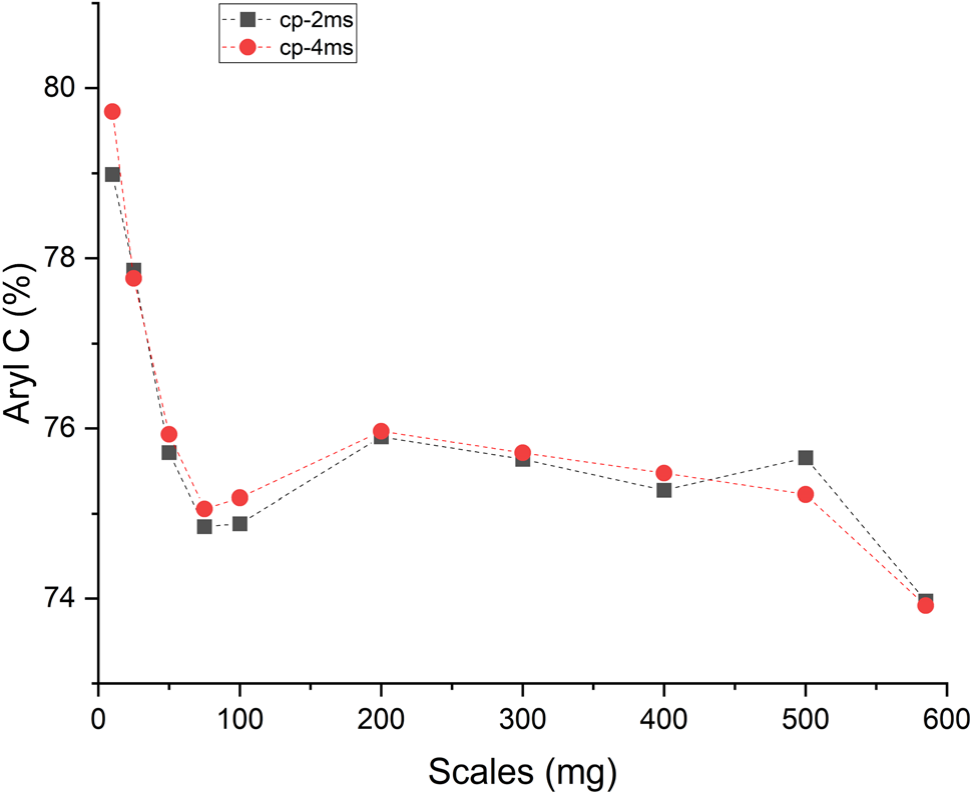
Amount of aryl carbon (% of total carbon) present in walnut shell biochar synthesized (in a thermogravimetric reactor) at different mass scales (mg) as determined from the ^13^C ssNMR crosspolarization (CP) measured with contact durations of 2 ms (cp-2 ms) and 4 ms (cp-4 ms).

In the Raman spectra (Fig. S4, ESI†) the D and G band are seen at ∼1356 and 1599 cm^−1^, respectively. The position of the G band at ∼1599 cm^−1^ does not change with scales and is similar to that of nanocrystalline graphite (nc-g) and biochar produced at HTT 700 °C.^70^ The blue shift of the G band (compared to that of graphite at 1580 cm^−1^) must be predominantly due to aromatic condensation^71^ rather than from the stress induced by the increased growth of polyaromatic structures into distorted graphene domains. The position of the dispersive D band is similar to that of non-graphitizing carbon seen in other studies^69,72^ using a 532 nm excitation laser. The broad peak of the second-order Raman band is similar to that of less ordered chars with s-ac as seen in other biochar synthesized at similar HTTs.^73^ The carbon structure of ws_scl lies between nc-g and s-ac in the amorphization trajectory of Ferrari *et al.*^74,75^Fig. 3 shows the evolution of *I*_D_/*I*_G_ and FWHM_G_ of the ws_scl, which are in close agreement between the two fitting methods. The decrease of the FWHM_G_ up to 100_scl is due to the decrease of the sp^2^ carbon (evident from the H/C trend in Fig. 1).^74^ The trend of the *I*_D_/*I*_G_ ratio shows that the defect density in the arrangement of BSUs increases up to 100_scl, after which it is fairly constant. Thus, the short-range order (*L*_a_) in s-ac seems to increase at low mass scales below 100 mg. This is also consistent with the increased H/C molar ratio at these small scales (Fig. 1) and the aryl-C% estimated using the ^13^C-ssNMR at CP at 2 ms and 4 ms of contact durations (Fig. 4). Thus, ws_scl carbon is predominantly composed of s-ac clusters (up to ∼80% aryl-C) with short-range defected nc-g with *L*_a_ ∼2 nm.

**Fig. 5:**
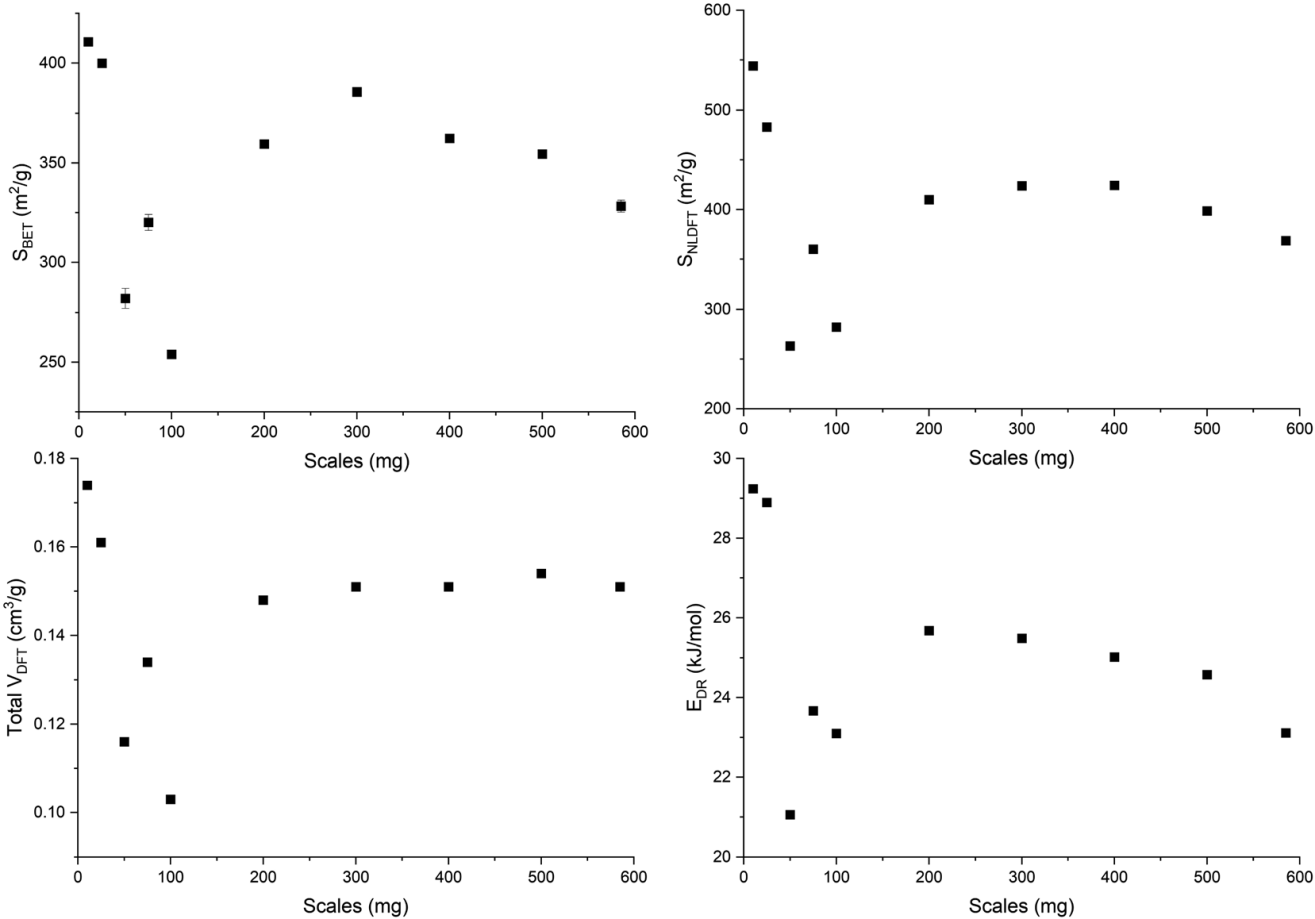
Pore surface area determined by BET (*S*_BET_), and 2D-NLDFT method (*S*_NLDFT_), total pore volume (*V*_DFT_), and characteristic adsorption energy (*E*_DR_) of the WS biochar derived at different mass scales (mg) during analytical pyrolysis in thermogravimetric reactor.

An open-loop hysteresis is present in the N_2_ and Ar isotherms due to the diffusion limitations during the desorption branch. Such open-loop hysteresis is not observed with CO_2_ due to its smaller kinetic diameter and the higher temperature at which the analysis is performed. The derived biochars are microporous (Fig. S5, ESI†). And Fig. 5 shows the microporosity – BET surface area (*S*_BET_), 2D-NLDFT surface area (*S*_NLDFT_), total pore volume (*V*_DFT_) – and the characteristic adsorption energy (*E*_DR_) of ws_scl. A trend is visible among the ws_scl with 50_scl as an outlier. The *S*_BET_, *S*_NLDFT_ and *V*_DFT_ are highest for small scales (10_scl, 25_scl), collapse at around 100_scl and then stabilize to an almost constant value at higher scales. This trend in microporosity is also reflected by the *E*_DR_. Non-graphitizing carbon derived from the pyrolysis of organic precursor usually shows a positive correlation with HTT up to an inflection point (∼700 °C). Then the pore area decreases at higher HTT. This phenomenon of pore collapse has been attributed to thermal deactivation (pore collapse, or stacking of short-range graphene sheets closing the pores and pore fusion)^36^ and to adsorption-induced pore collapse during N_2_ physisorption (a commonly used Porosimetry technique for biochars) in a recent study by Maziarka *et al.*^39^ Here, although the HTT is invariant, there is a collapse in microporosity at 100_scl. The collapse is due to a decrease in the short-range graphene layers or structural order of carbon (srso) from 10_scl to 100_scl as seen in Fig. 3. Then, srso remains fairly constant from 200_scl, but with an increased pore surface area and pore volume compared to 100_scl. This increase is not due to pore widening, since the ratio of micro-to mesopores is relatively equal (Fig. S5, ESI†). The presence of alkali and alkaline earth metals (AAEM), P and S is also not responsible for this increase^76^ since the concentrations of these elements have not varied significantly in the derived biochar as seen in Fig. S6, ESI.† Activation during pyrolysis in the presence of evolved CO_2_ at these mass scales may be a potential explanation.^77^ However, this requires further investigation.

The structural development models of non-graphitizing carbon here are consistent with those of Bourke *et al.*^66^ and McDonald-Wharry *et al.*^63^ The extent to which covalently bonded cross-links in ws_scl lead to pentagonal and heptagonal rings requires future investigation using transmission electron microscopy.^61^ It is also hypothesized that further heating of the biochar derived at smaller scales, (such as 10_scl and 25_scl), to temperatures >2000 °C would result in relatively more graphitic stacking than those at the higher scales due to higher heteroatom-induced kinetic locking or srso topological defects in the latter.

### 4.2 Influence of scaling on pyrolysis characteristics

It is worth mentioning at the beginning of this section that slow pyrolysis is generally defined as a thermal treatment in an inert atmosphere where the heating rate is between 6–48 °C min^−1^,^78^ 0.6–120 °C min^−1^ (ref. 79) or 6–60 °C min^−1^ (ref. 23) and fast pyrolysis can have higher heating rates up to 6000 °C min^−1^.^80^ Such variations in heating rates in this literature definition arise from the reasoning that pyrolysis prefers the char formation route when the heating time is less than the pyrolysis reaction time.^81^ For example, in Fig. S7 (ESI†), even at a heating rate of 100 °C min^−1^, the WS undergoes slow pyrolysis (HTT of 650 °C) with only a slightly higher yield compared to 3 °C min^−1^. This is because sample mass, particle size, and reactor configuration are also critical parameters that determine the heating rate beyond which “slow” transitions to fast pyrolysis. It is recommended that such definitions be carefully worded in future literature reviews.

From the thermograms (Fig. S8–S9, ESI†), WS volatilizes ∼70% of its initial weight before 400 °C in open crucibles and pl_crucibles, completing the primary devolatilization stage (PVS). Temperatures above 400 °C would consist mainly of polycondensation, arrangement of BSUs, cross-linking by heteroatoms, and devolatilization of recalcitrant lignin. The poor *λ* of the biochar–biomass mixture resulted in the shift of the maximum peak decomposition temperature (MPT) from 325 to 375 °C as *β* increased from 3 to 20 °C min^−1^. The heat flow during PVS is predominantly exothermic in pl_crucibles. This is because heterogeneous (solid–gas) secondary reactions (HeSTR) are favoured in pl_crucibles due to the longer contact time of the gaseous tar with the solid char which leads to higher biochar yields during PVS. This is also supported by other pyrolysis studies.^46,82^ The *E*_*α*_ and ln *A* during PVS, *α* = 0.3 to 0.65, (Fig. S10, ESI†), are also consistent with similar biomass pyrolysis^83^ and show minimal variation, indicating that there may not be multiple steps involved in the overall process kinetics during this stage.^84^ Beyond *α* = 0.7, the sharp increases in *E*_*α*_ and ln *A* mark the final stages of PVS. The HeSTR also makes the pyrolysis in pl_crucibles more favourable (lower Δ*G*_*α*_). Therefore, the scaling experiments were performed only with open-lid crucibles to isolate the HeSTR caused by increasing sample masses from that caused by the crucible.

**Fig. 6:**
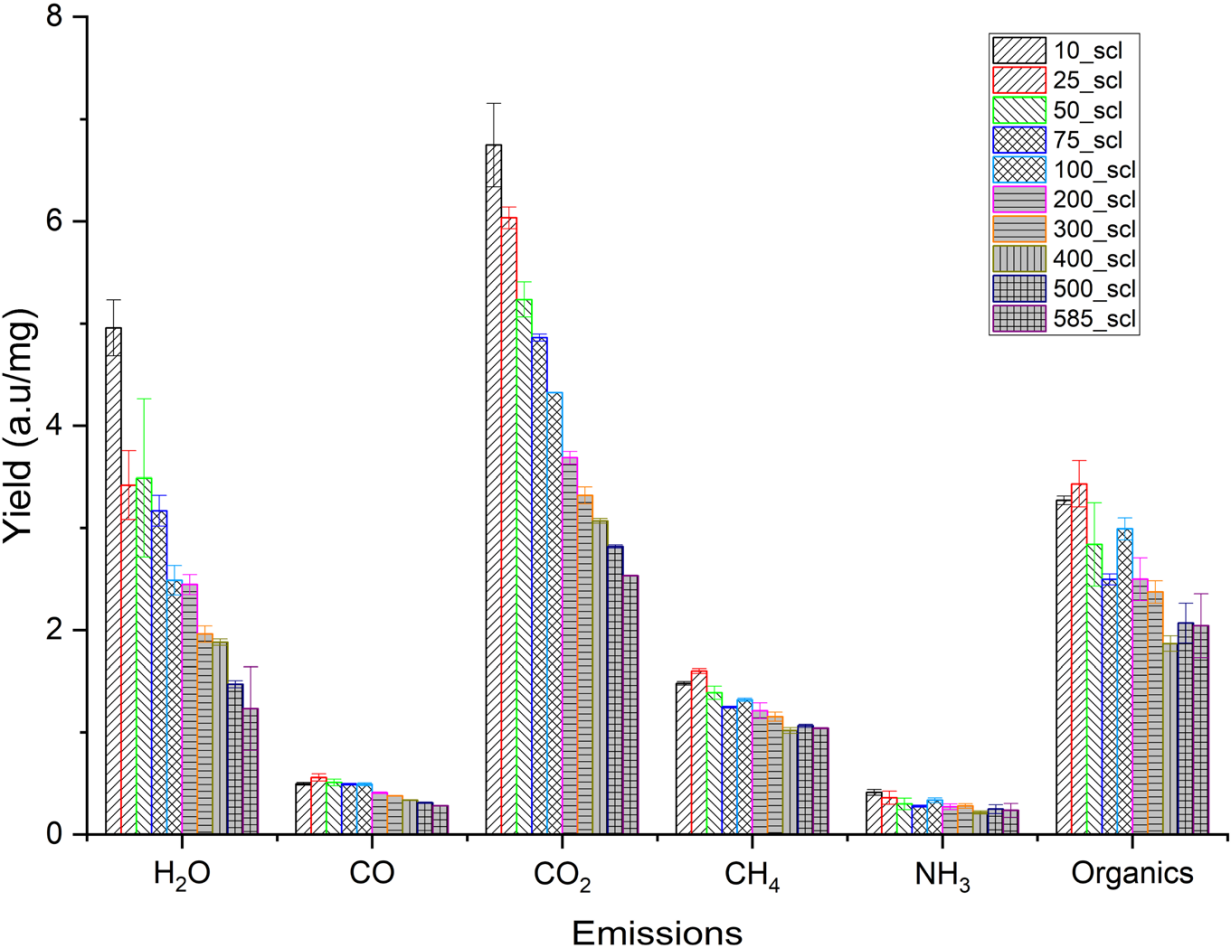
Total yield (normalized by mass loss) of different emissions during the pyrolysis of walnut shell powder at different mass scales (scl) in thermogravimetric analyzer coupled with an online FT-IR. Note: 10_scl, 25_scl *etc.* Refers to mass scale of 10 mg, 25 mg, respectively.

The relative emission intensities during scaling are shown in Fig. 6. CO_2_ and H_2_O originate from decarboxylation and dehydration, respectively. CH_4_ can originate from the cracking of methoxy groups and the fragmentation of the lignin side chains.^85^ The emission intensities of CO_2_, H_2_O and CH_4_ decrease with increasing scale, confirming the trend of higher char formation and biochar yield at higher mass scales that was seen previously. The release profile of NH_3_, an indicator of N distribution in the emissions for lignin-rich substrates,^86^ shows no statistically significant variation. Furthermore, the evolution of this NO_*x*_ precursor is not predominantly due to the conversion of N present in biomass,^87^ and attempts to investigate the N distribution during biomass pyrolysis are still ongoing.^86^

**Fig. 7:**
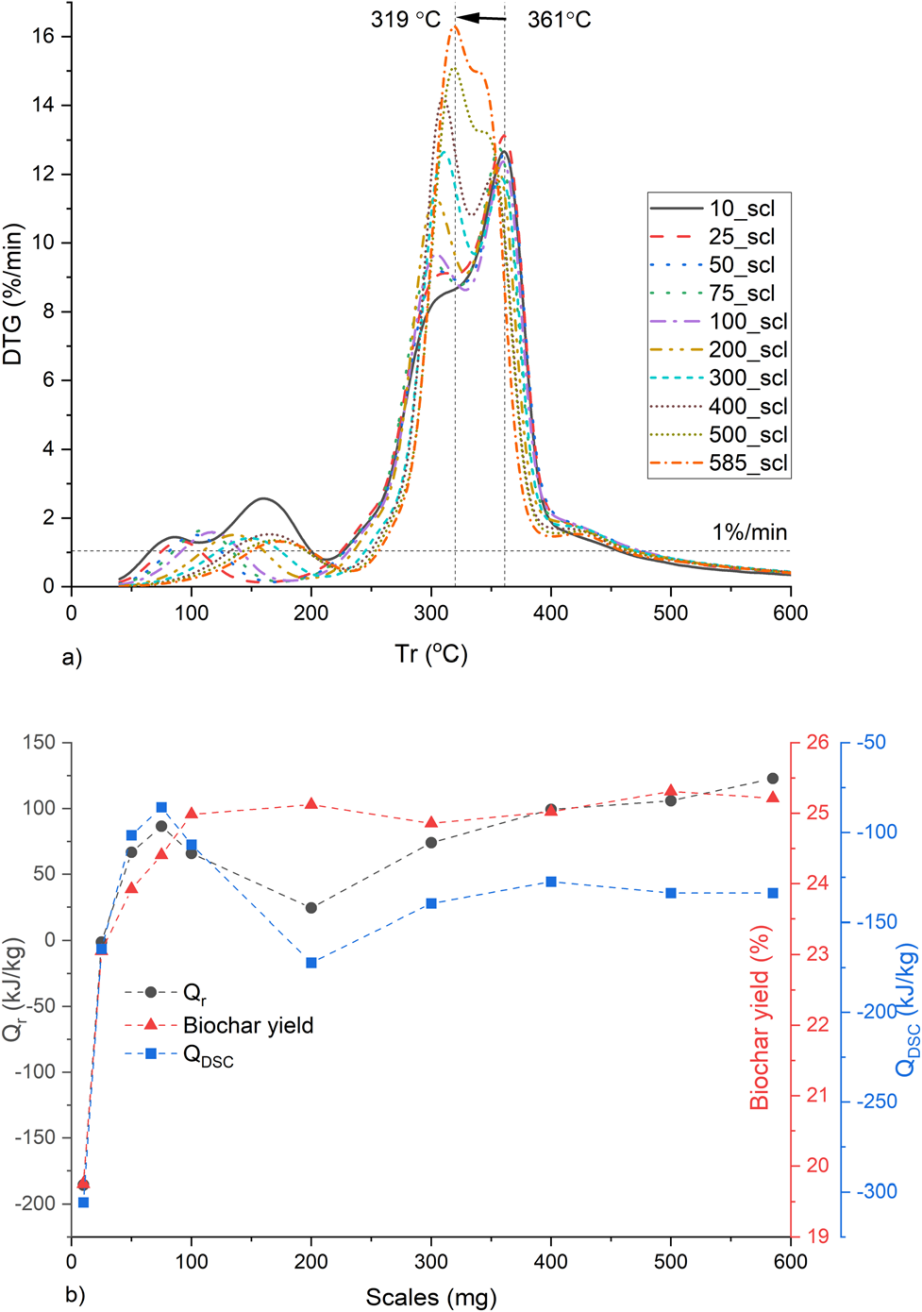
Biochar synthesis at different mass scales (mg) in a thermogravimetric reactor showing (a) rate of change of mass with respect to furnace temperature (*T*_r_), (b) heat of reaction (*Q*_r_), biochar yield and total heat flow (*Q*_DSC_) between 60 and 554 °C. Note: 10_scl, 25_scl *etc.* Refers to mass scale of 10 mg, 25 mg, respectively.

The *Q*_rad_ is prominent at temperatures above ∼554 °C as seen in Fig. S11 (ESI†). And the DSC sensor requires temperatures up to 60 °C for signal stabilization. Therefore, *Q*_r_ and *Q*_dsc_ were calculated between 60 and 554 °C with an end-to-end baseline correction. The MPT gradually shifts from 361 to 319 °C with increasing mass scales (Fig. 7a). The total *Q*_r_ and *Q*_DSC_ increase up to 75_scl, then decrease and maintain similar values beyond 200_scl (Fig. 7b). The shift in the MPT at higher scales is due to the HeSTR, which increases the exothermicity of the reactions and thereby increases the reaction rate at lower temperatures due to localized overheating. This was also observed in the case of Ba(TFA)_2_.^88^ The dip in *Q*_dsc_ after 75_scl may be due to the endothermic tar cracking HoSTR, which is also promoted as the localized overheated zones approach 500 °C. This would also be evident from a corresponding increase in CO, which is visible here at ∼20 min (Fig. S12, ESI†).^89^ At ∼20 min, there is an increase in CH_4_ (Fig. S13, ESI†) for scales above 200_scl. These are also plausible indicators that above 200_scl, CO_2_ is trapped within the core of the substrate, initiating more CO and CH_4_ formation through the thermal cracking of tar and the Boudouard reaction (CO_2_ + C → 2CO).^90^ Such cracking may also have caused the increase in *S*_NLDFT_ from 200_scl, similar to the report of Greco *et al.*^91^ Another interesting finding is that, for open crucibles and pl_crucibles, the scales from 75_scl show similar *Q*_DSC_ (Fig. S14, ESI†). Below 75_scl, the pyrolysis in 900 μl open crucibles is more exothermic than the 900 μl pl_crucibles, which is in contrast to the case of 70 μl crucibles (described previously, and also seen in other investigations^46^). This is because the 900 μl crucibles have *h*_c_ ∼ 1 cm, compared to the 0.44 cm of the 70 μl crucibles. This may give the evolved gases more time in the 900 μl crucible before being purged from the furnace environment, resulting in more cracking of volatiles. However, at higher scales, the substrate height increases relative to *h*_c_, the transfer time decreases, HeSTR increases, and the *Q*_DSC_ of open and pl_crucibles become similar.

For the WS precursor, scales above 200 mg can be used for the synthesis of biochar in a TG reactor since scaling effects on biochar properties and pyrolysis characteristics are minimized beyond this inflection mass. A similar inflection point was also seen when a cellulose-rich banana peduncle (BP) was used as the substrate in the authors' preliminary study.^92^ However, the BP was rich (6.53 dry wt%) in potassium,^93^ an AAEM that catalyzes pyrolysis making it comparatively difficult to isolate scaling from AAEM influence as a cause of the scaling trend and the inflection point. Hence, such scaling studies are better performed with relatively pure (minimal AAEM and transition metals) biomass such as walnut shells^94^ or wood after a comprehensive fiber and elemental analysis. For WS, the H/C molar ratio of ws_scl until 200 mg scale shows a strong correlation with (a) biochar properties of aryl-C% (*r* = −0.972, *p* = 0.006), surface area (*r* = −0.971, *p* = 0.028), (b) the pyrolysis *Q*_DSC_ (*r* = +0.981, *p* = 0.003), and (c) CO_2_ emission (*r* = −0.979, *p* < 0.004). If these scaling correlations of H/C with biochar and pyrolysis properties can be extended to more types of lignin-rich precursors (such as wood, pinecones, *etc.*) and include additional intermediate mass scales (such as 150, 250 mg, *etc.*), then it will be possible to generalize scaling effects to a precursor based on its properties such as aspect ratio, and elemental concentrations of CHNO.

## 5. Conclusion

The influence of sample mass (scaling effect) on the synthesis and structure of non-graphitizing carbon derived from the analytical pyrolysis of biomass (in TG reactor) has been identified, traced, and comprehensively investigated with the example of a walnut shell substrate. The carbon in ws_scl is sp^2^-amorphous with short-range defected nanocrystalline graphite. It is demonstrated that scaling affects the pyrolysis process and the properties of the resulting biochar carbon. The effect of scaling is seen as a gradual shift in the process characteristics and the properties of the biochar carbon until it reaches an inflection point. After this inflection point of ∼200 mg (for the WS precursor used here), the aryl-C, pore characteristics, defects in the carbon network, *Q*_DSC_, and biochar yield are quite similar. Moving from small scales (∼100 mg) towards the pure kinetic regime (≤10 mg), the carbonization increases, and the pyrolysis becomes more endothermic with a decrease in HeSTR and increased emissions (CO_2_ and H_2_O). The determination of heat of pyrolysis (*Q*_DSC_ or *Q*_r_) is sufficient to semi-quantitatively determine the inflection point since it shows a strong correlation with the carbonization of resultant biomass. Thus, for lignin-rich substrates such as WS, TG reactors can be used for concurrent process characterization and biochar synthesis (for investigating the NGC) after the estimation of the inflection mass scale based on *Q*_DSC_ and/or H/C of the biochar. It can be concluded that there is a potential for utilizing TG reactors in the rapid prototyping and improving the technology readiness level of application-specific biochar design to level 6 or higher.^23^ However, the limitation of this study is that it has not explained a generalized mechanism behind the influence of mass scaling (in the TG reactor). The main cause of the scaling effect may be the combined effect of the thermal history of the sample during the C1 carbonization stage on the C2 stage, and the char (s)–tar (g) reactions in the crucibles with higher surface area (that influences the HeSTR and HoSTR above 600 °C) used in macro-TG systems. To confirm this, more biomass types must be subjected to scaling investigations with the properties of NGC also studied at multiple stepwise increments of temperature (say, 100 °C) until the HTT. This opens an avenue for follow-up research that has the potential to advance the methodology of lab-scale biochar synthesis.

## Author contributions

Conceptualization and planning – R. R. N., D. W.; experiments – R. R. N., P. A. K.; methodology – R. R. N., P. A. K., M. S.; NMR analysis – A. M., R. R. N.; data curation & visualization: R. R. N.; formal analysis: R. R. N.; writing (original draft) – R. R. N.; writing (review and editing): R. R. N., A. S., D. W.; supervision: D. W., P. B.; funding & resources: D. W., P. B., M. S., A. M.; project administration: D. W., P. B.; all authors have read and agreed to the published version of the manuscript.

## Conflicts of interest

The authors declare no financial or any other form of competing interests in this article.

## Supplementary Material

RA-013-D3RA01911J-s001
