# Supplementary material for: The influence of sample mass (scaling effect) on the synthesis and structure of non-graphitizing carbon (biochar) during the analytical pyrolysis of biomass
Source: RSC Adv. 2023 May 2;13(20):13526–39. doi: 10.1039/d3ra01911j (PMC10153483; doi:10.1039/d3ra01911j)
Supplement: RA-013-D3RA01911J-s001 [file RA-013-D3RA01911J-s001.pdf]

Electronic Supplementary Material (ESI) for RSC Advances.
This journal is © The Royal Society of Chemistry 2023

Supplementary Information

The influence of sample mass (scaling effect) on the synthesis and structure of non-graphitizing carbon (biochar) during the analytical pyrolysis of biomass

Rahul Ramesh Nair [a*], Patrick A. Kißling [b], Andreas Schaate [c,d], Alexander Marchanka [e], Madina Shamsuyeva [f], Peter Behrens† [c,g], Dirk Weichgrebe [a]

[a] Institute of Sanitary Engineering and Waste Management (ISAH), Leibniz University of Hannover, Hannover, 30167 Germany

[b] Institute of Physical Chemistry and Electrochemistry (PCI), Leibniz University of Hannover, Hannover, 30167 Germany

[c] Institute of Inorganic Chemistry (ACI), Leibniz University of Hannover, Hannover, 30167 Germany

[d] Laboratory of Nano and Quantum Engineering, Leibniz University of Hannover, Hannover, 30167 Germany

[e] Institute of Organic Chemistry and Centre of Biomolecular Drug Research (BMWZ), Leibniz University of Hannover, Hannover, 30167 Germany

[f] Institute of Plastics and Circular Economy (IKK), Leibniz University of Hannover, Garbsen, 30823 Germany

[g] Cluster of Excellence PhoenixD (Photonics, Optics, and Engineering – Innovation Across Disciplines), Leibniz University of Hannover , Hannover, 30167 Germany

* Corresponding author, Email: nair@isah.uni-hannover.de, rahulgenesis@hotmail.com

In memory of Professor Dr. Peter Behrens

Contents

1. Properties of walnut shell powder 2
1.1 Particle size distribution 2
1.2 Density and thermal conductivity 2
1.3 Specific heat capacities 3
2. Results 4
2.1 Thermal recalcitrance 4
2.2 Raman spectra 4
2.3 Pore size distribution 5
2.4 Slow pyrolysis of biomass 8

1. Properties of walnut shell powder

1.1 Particle size distribution

The measurement was carried out using a static approach with PowderShape FH with Add-on FibreShape (IST Innovative Scan Technologies AG, Flensburg, Germany). Before the measurement the sample container is slightly shaken so that the particles are uniformly distributed within the container. Afterwards, a small amount of the powder is distributed on a A4 scanner and measured with a resolution of 1600 dpi. The number of measured objects (particles) was 68,761. The area occupied by the objects was 5.4% of the scanner area.

1.2 Density and thermal conductivity

The thermal conductivity of walnut shell powder is λ = 79.82 mw m^{-1} K^{-1}) at 40 °C, and was estimated by Lambda-Messtechnik GmbH (Dresden, Germany) using lambda-Meter EP500e according to EN 1946-2. The density of walnut shell powder is ρ = 511.32 kg m^3.

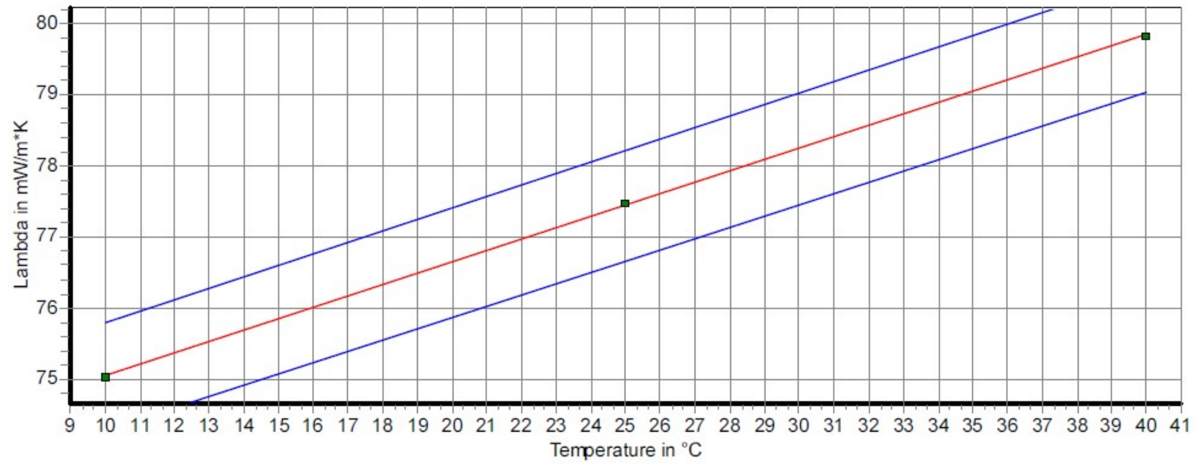

Figure S1: Three-point thermal conductivity λ (mW/m·K) measurement at temperatures of 10, 25 and 40 °C according to EN 1946-2. λ = f(T) = 0.1593 * T + 73.46, where T is temperature of the walnut shell powder in °C

1.3 Specific heat capacities

The specific heat capacities of biomass $c_{p,b}$ (J/(g·K)) and char $c_{p,c}$ (J/(g·K)) were evaluated using a (Erich Netzsch GmbH & Co. Holding KG, Germany) with sample mass of 2 ± 0.2 mg in Aluminum Concavus pans with lids. Their temperature-dependent relationship is shown in Figure S2.

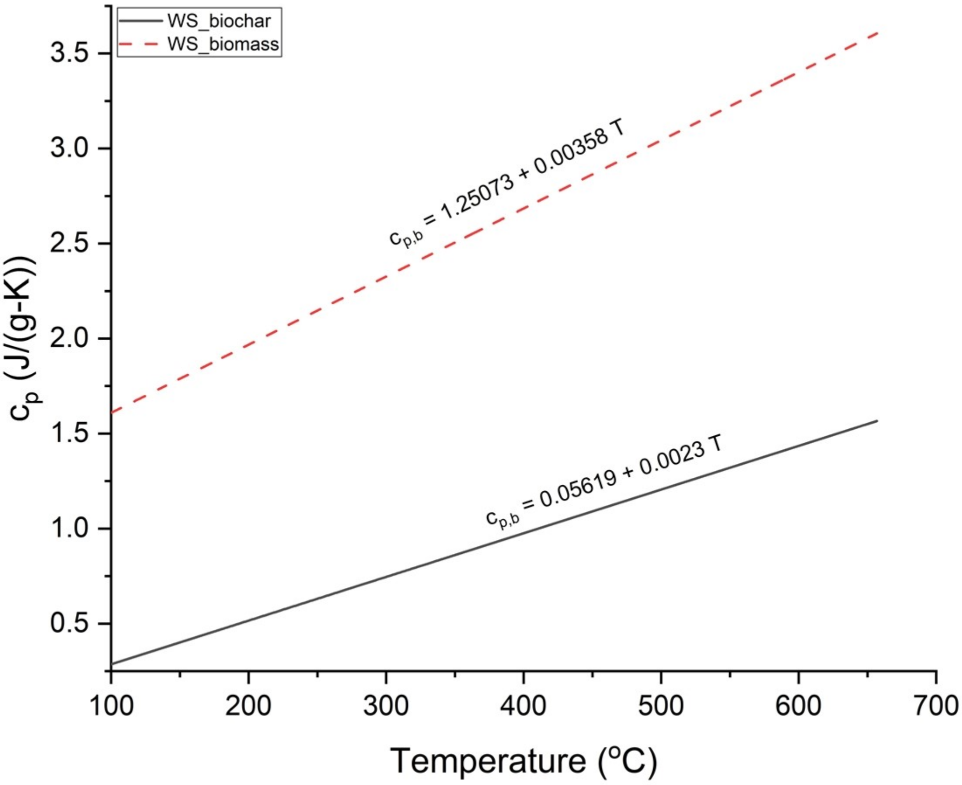

Figure S2: The temperature (T) dependence of specific heat capacities of walnut shell (WS) powder $c_{p,b}$ (J/(g·K)) of and its biochar $c_{p,c}$ (J/(g·K)) evaluated using differential scanning calorimetry (DSC). The particle size distribution and bulk density of WS are 129 ± 115 μm, 511.32 kg/m³, respectively.

2. Results

2.1 Thermal recalcitrance

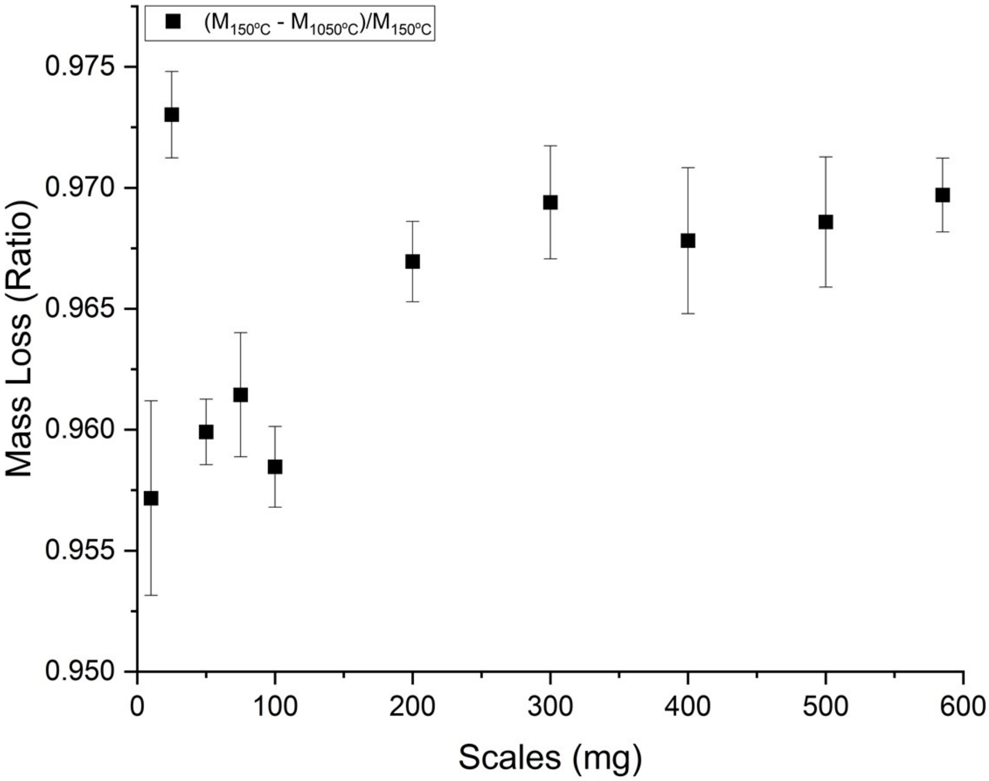

Figure S3: Thermal recalcitrance (expressed as mass loss ratio) of the walnut shell biochars prepared at different mass scales (mg) in the TG reactor expressed as mass loss ratio (M) between 150 and 1050 °C. The scale of 25 mg seems to be an outlier.

2.2 Raman spectra

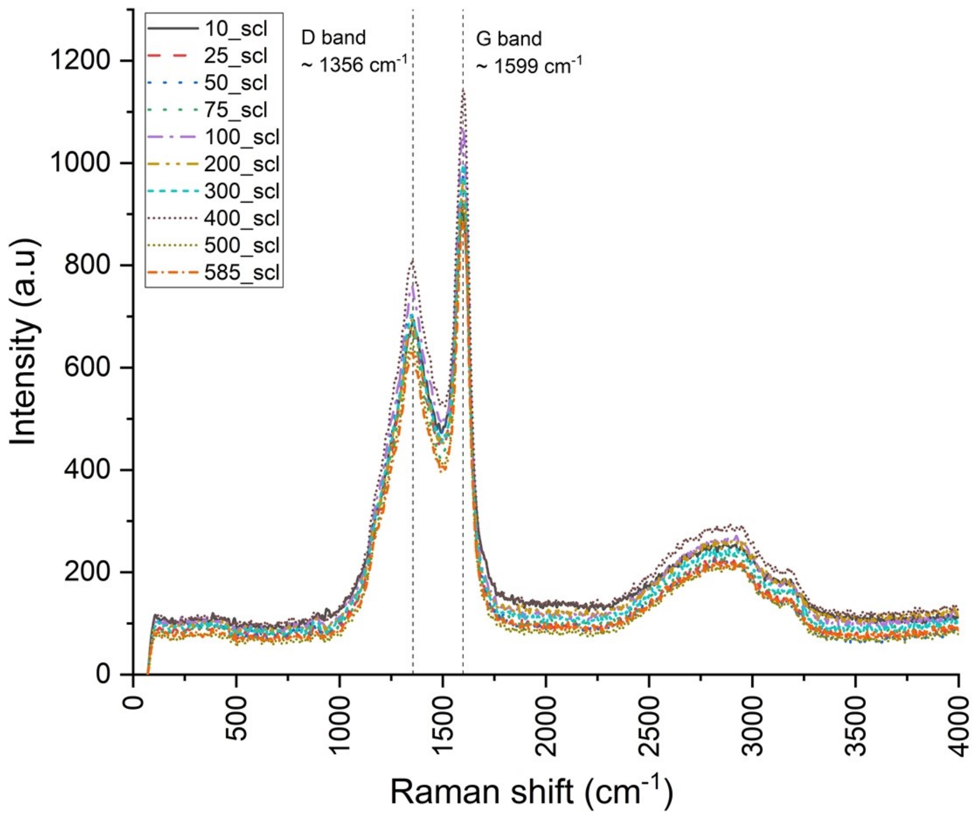

Figure S4: Unprocessed Raman spectra of biochar derived from walnut shells at different scales in a Thermogravimetric reactor. Note: 10_scl, 25_scl etc. stands for the mass scale of 10 mg, 25 mg, respectively.

2.3 Pore size distribution

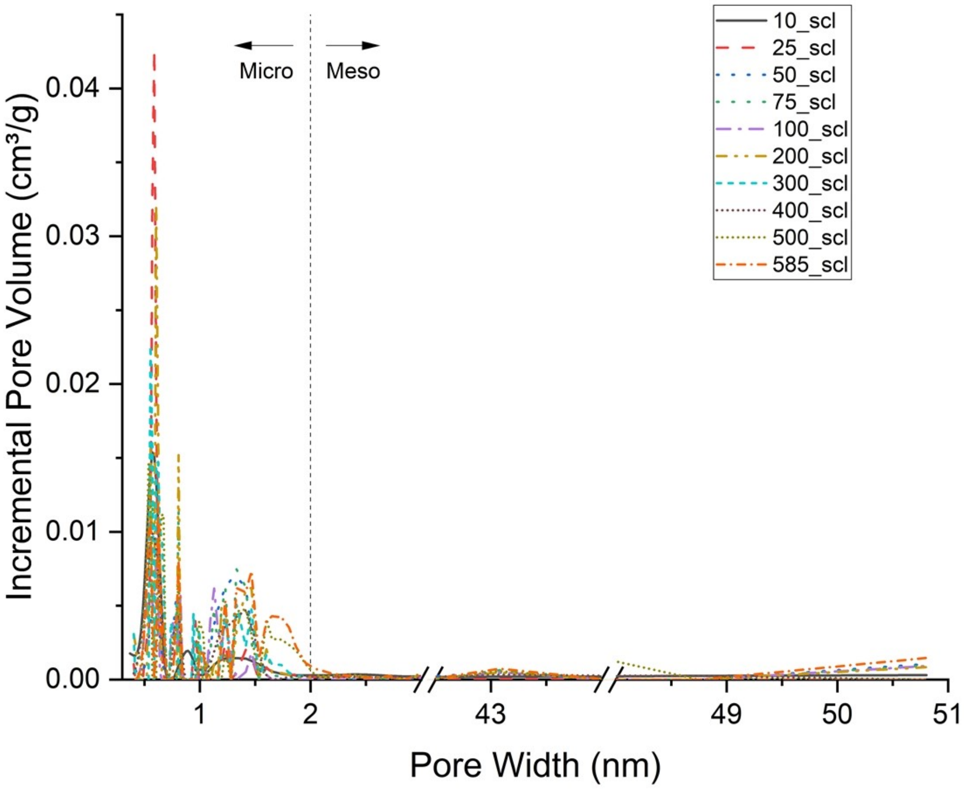

Figure S5: Pore size distribution of the walnut shell biochar derived at different scales (scl) evaluated using Ar physisorption (using method of HS-2DNLDFT). Note: 10_scl, 25_scl etc. stands for the mass scale of 10 mg, 25 mg, respectively.

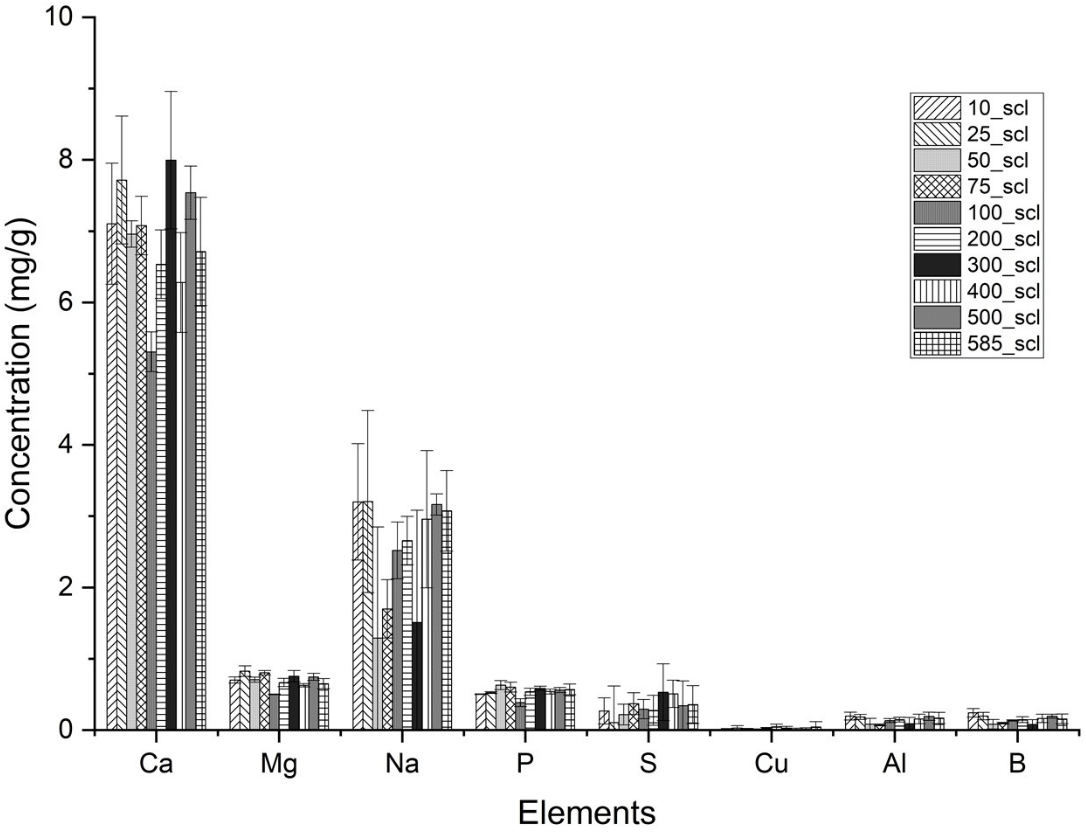

Figure S6: Concentration of elements (mg/g) present in Walnut shell biochar prepared at different mass scales between 10 mg (10_scl) and 585 mg (585 _scl) in TG reactor.

2.4 Slow pyrolysis of biomass

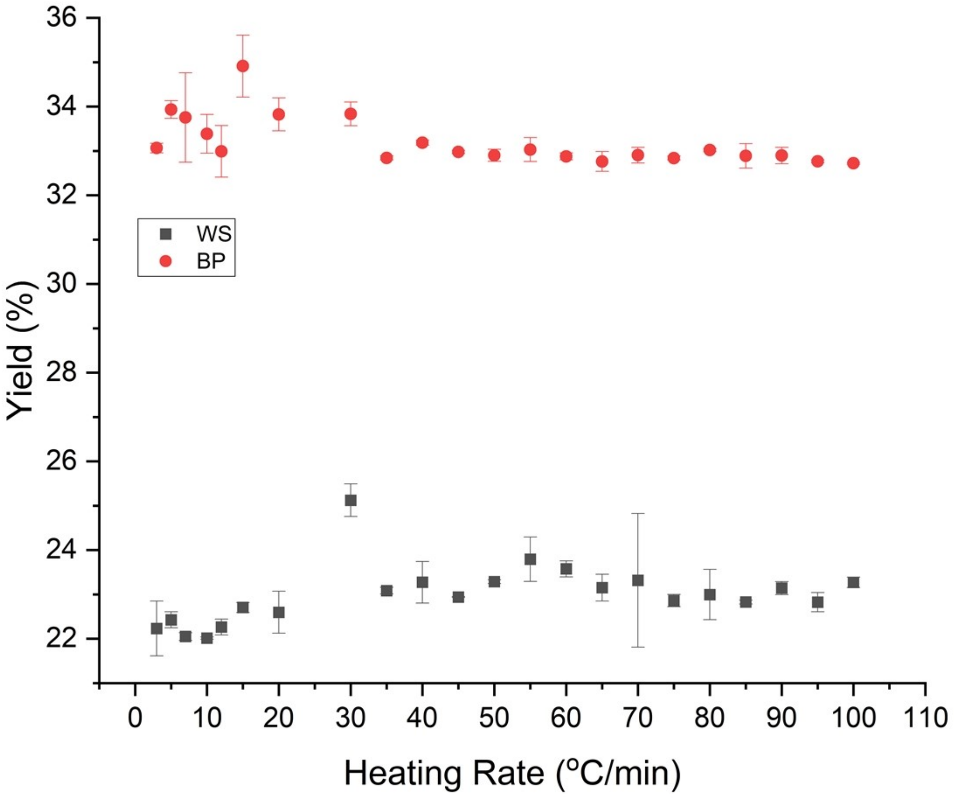

Figure S7: Biochar yield (%) during the slow pyrolysis of walnut shell (WS) powder (0.2mm, 5 mg) and banana peduncle (BP), (0.2mm, 10 mg) at heating rates between 3 to 100 °C in a TG reactor. The highest treatment temperature is 650 °C. The pyrolysis of BP is used for comparison of cellulose-rich to a lignin-rich feedstock such as WS.

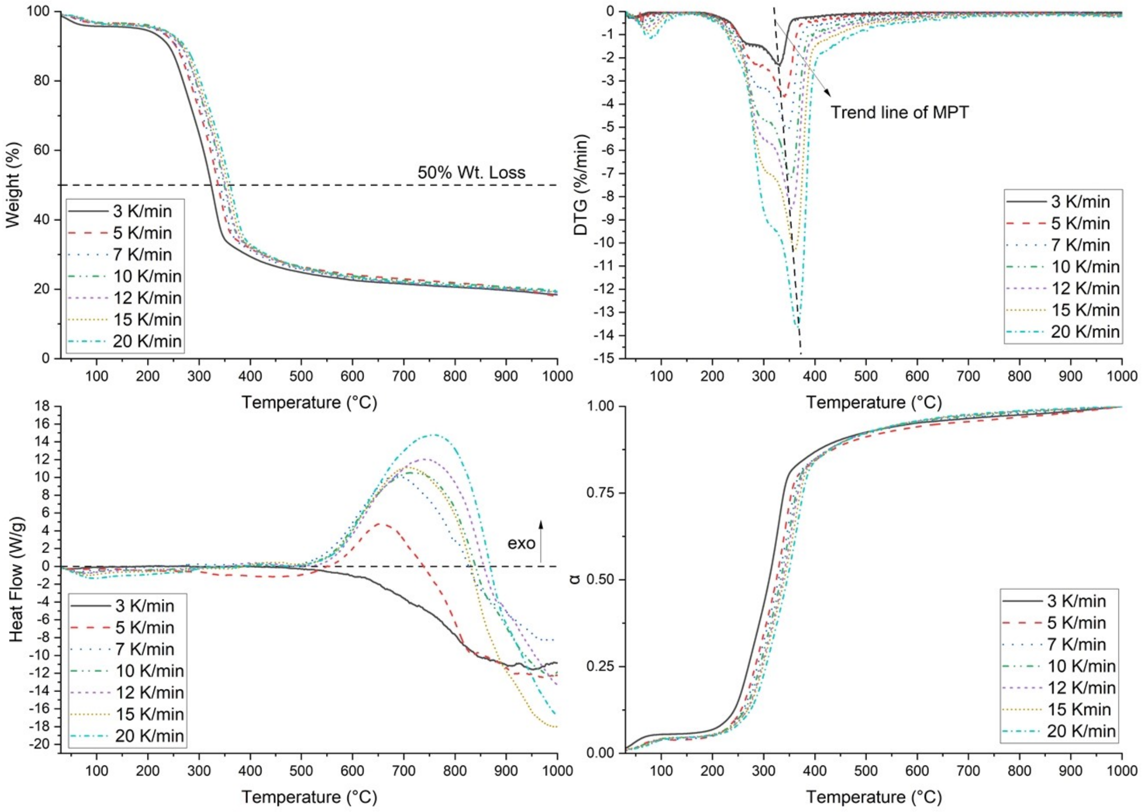

Figure S8: Thermograms – weight (%), rate of change in weight, DTG, (%/min) and heat flow (W/g) – and conversions (α) of walnut shell powder (5 mm) during pyrolysis at 6 different heating rates without any lid covering the crucible.

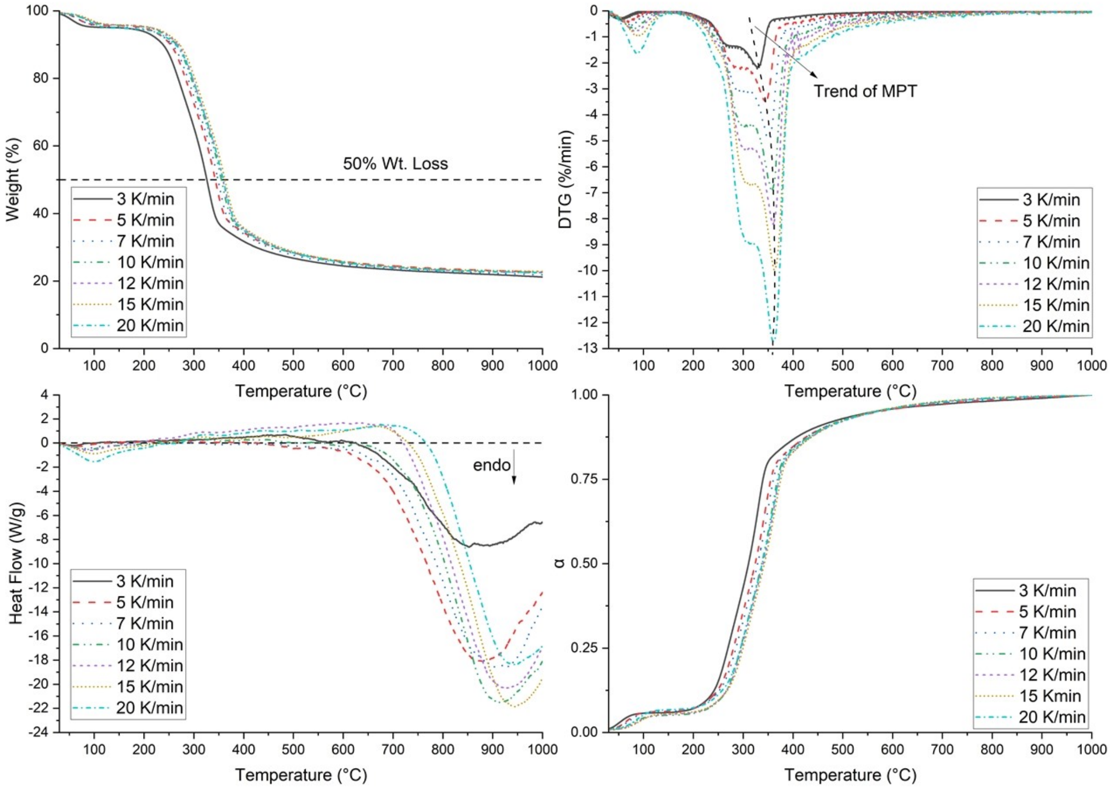

Figure S9: Thermograms – weight (%), rate of weight change, DTG, (%/min) and heat flow (W/g) – and conversions (α) of walnut shell powder (5 mg) during pyrolysis (in TG reactor) at 6 different heating rates with a pierced lid covering the crucible.

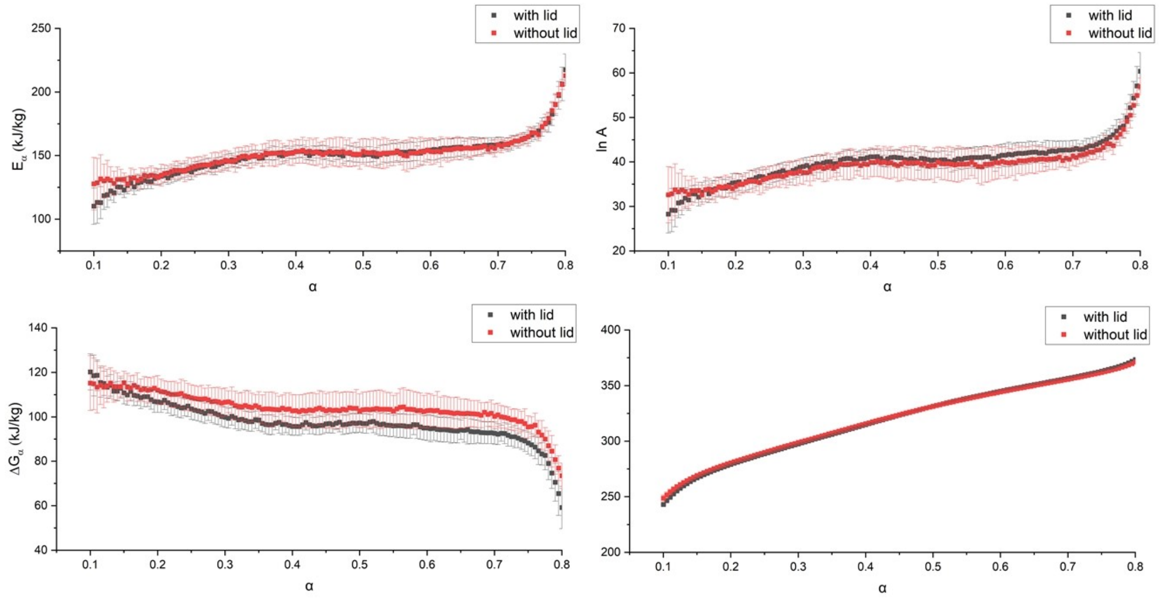

Figure S10: Conversion (α) dependent activation energy, E_α, (kJ/kg) pre-exponential factor (ln A), Gibbs free energy, ΔG_α, (kJ/kg) evaluated from the kinetic analysis during the slow pyrolysis of walnut

shell powder using 70 µl alumina crucibles that have open-lid and pierced-lid configurations. The changes in crucible temperature with α (bottom right) is also shown for reference.

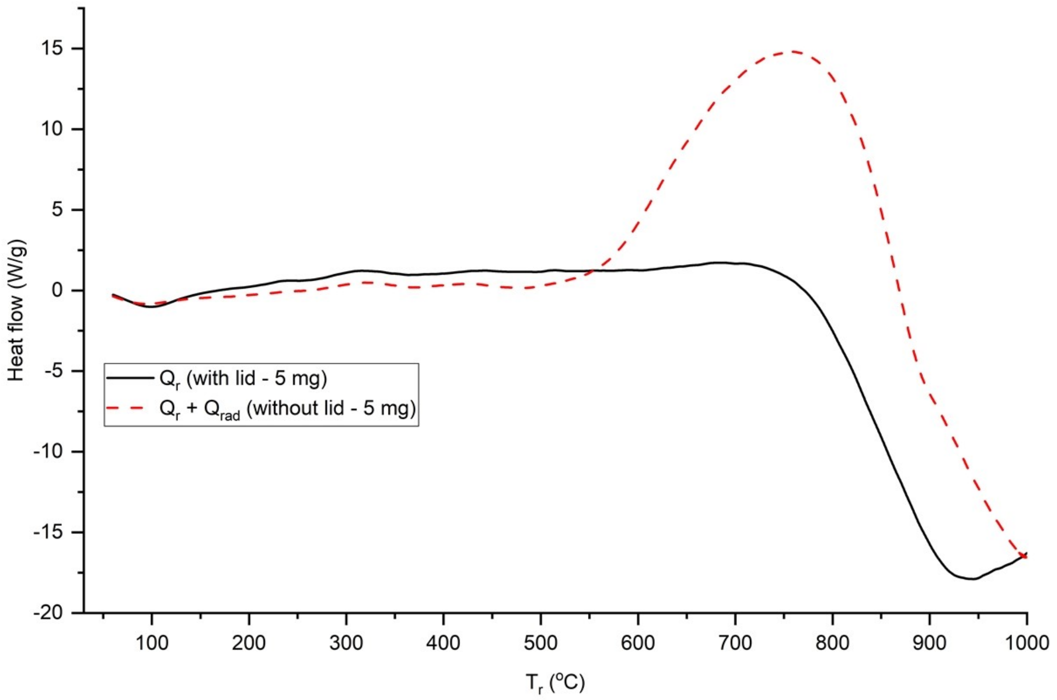

Figure S11: Heat flow (W/g) vs reference temperature, T_r, (°C) during the pyrolysis of walnut shell powder (5 mg) in a TG reactor under the pure kinetic regime in alumina crucibles with and without pierced-lid. Q_r is the heat of reaction and Q_{rad} is the radiative heat flow between the char and furnace in crucibles having no lid. Negative heat flow is endothermic in nature.

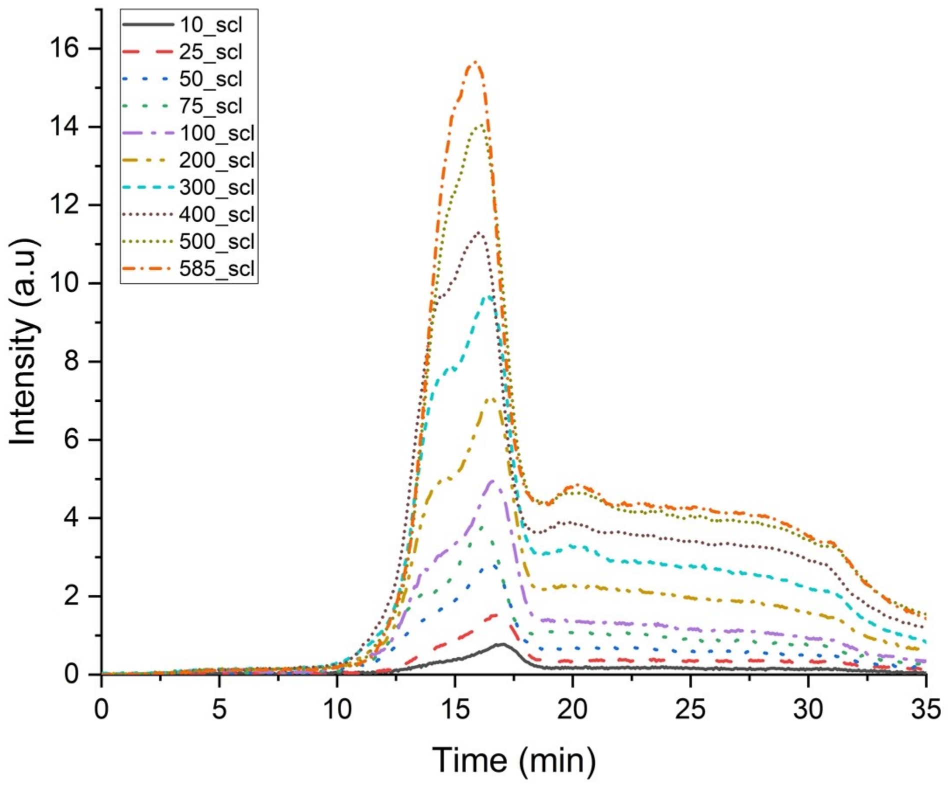

Figure S12: Evolution profile (non-normalized) of CO for the walnut shell pyrolysis (25 to 650 °C at heating rate 20 °C/min) using different sample mass scales (scl) plotted against time. Note: 10_scl, 25_scl etc. stands for the mass scale of 10 mg, 25 mg, respectively.

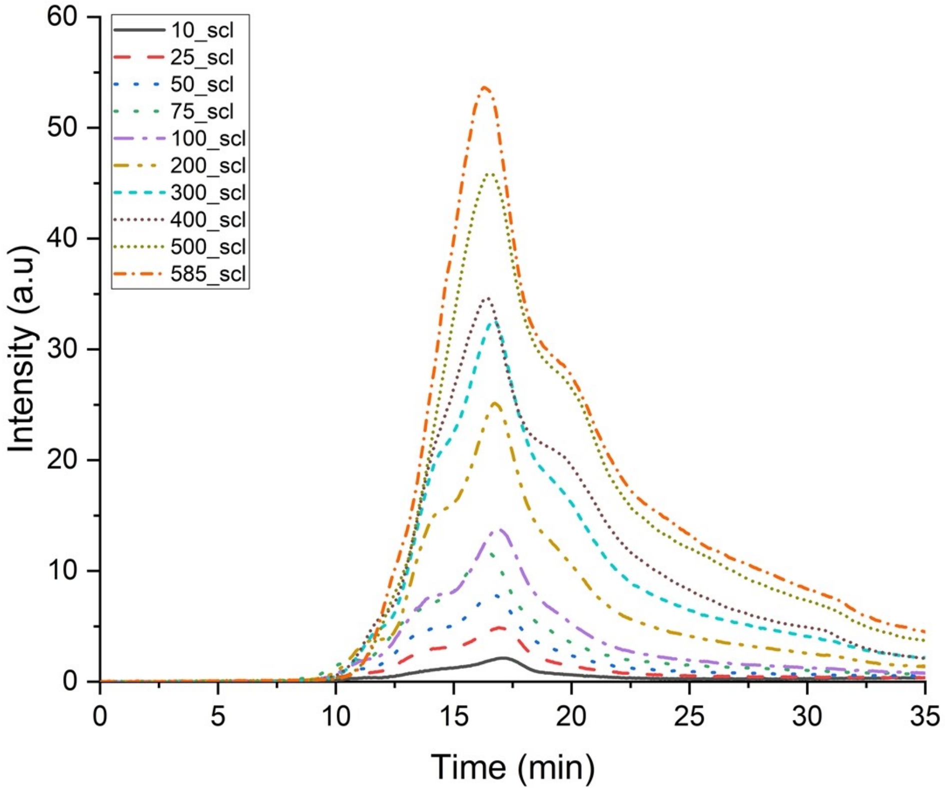

Figure S13: Evolution profile (non-normalized) of CH_4 for the walnut shell pyrolysis (25 to 650 °C at heating rate 20 °C/min) using different sample mass scales (scl) plotted against time. Note: 10_scl, 25_scl etc. stands for mass scale of 10 mg, 25 mg, respectively.

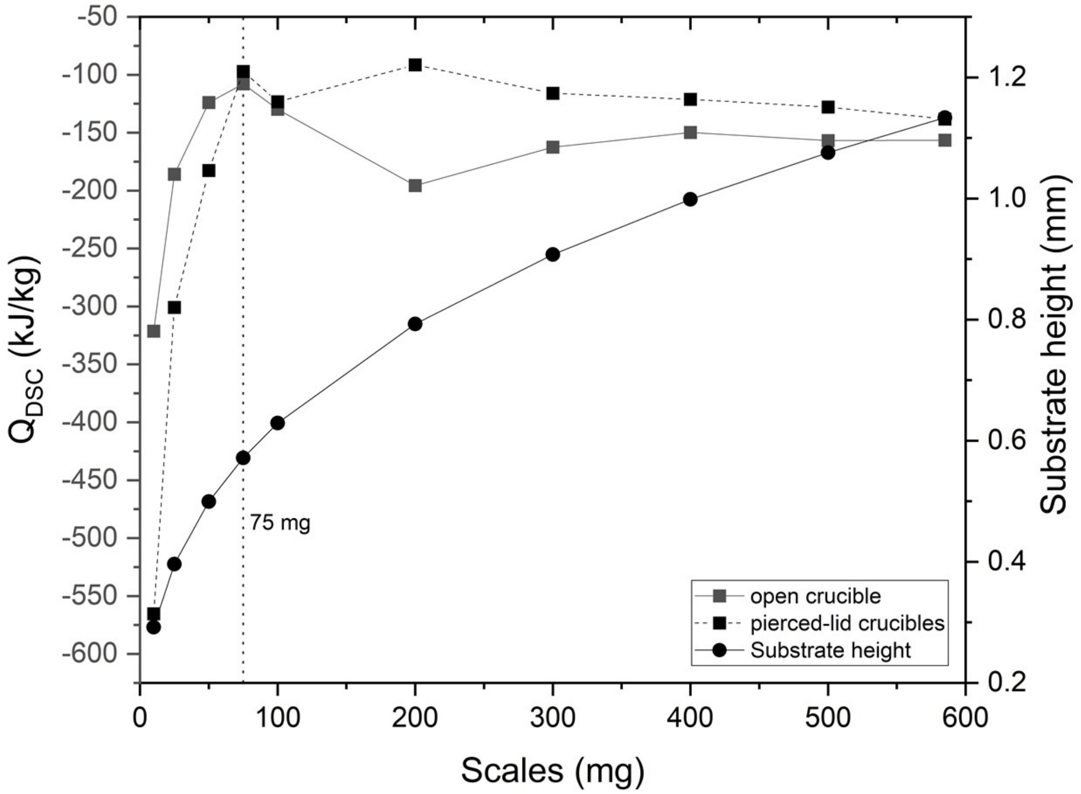

Figure S14: Heat flow, Q_{DSC}, (kJ/kg) during the synthesis of WS biochar (left-Y) at different mass scales (in mg) during analytical pyrolysis in a thermogravimetric reactor using alumina crucibles without lid (open) and pierced-lid. The change in substrate height (right-Y) with the increase in mass scales is also shown. Negative Q_{DSC} denotes endothermicity (heat required).
